# Assessing the Health Impact of Disinfection Byproducts in Drinking Water

**DOI:** 10.1021/acsestwater.3c00664

**Published:** 2024-03-02

**Authors:** Indrajit Kalita, Andreas Kamilaris, Paul Havinga, Igor Reva

**Affiliations:** † Computing & Data Sciences (CDS), Boston University, Boston, Massachusetts 02215, United States; ‡ CYENS Centre of Excellence, Nicosia 1016, Cyprus; § Pervasive Systems Group, University of Twente, Enschede 7522, Netherlands; ∥ Department of Chemical Engineering, CERES, University of Coimbra, Coimbra 3030-790, Portugal

**Keywords:** disinfection byproducts, drinking water quality, human health impact, natural organic matter, environmental conditions

## Abstract

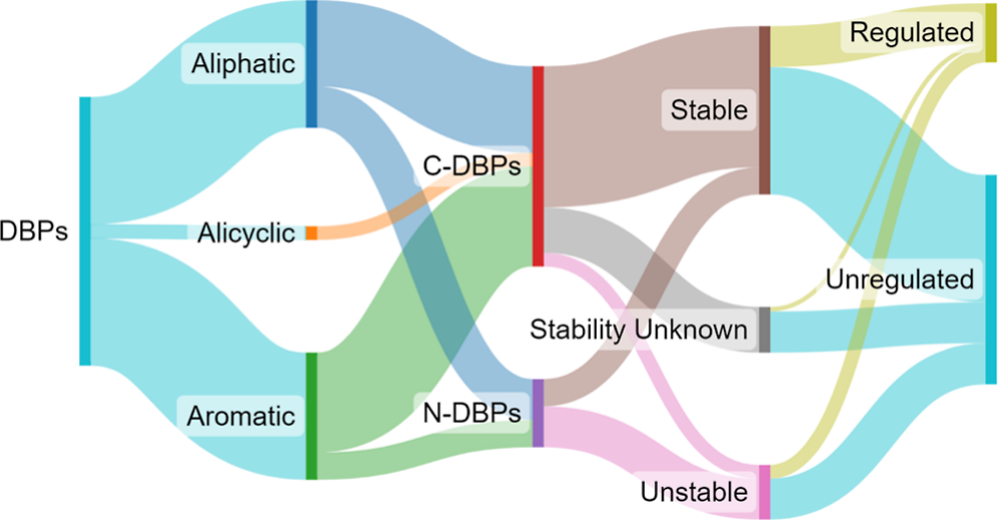

This study provides a comprehensive investigation of the impact of disinfection byproducts (DBPs) on human health, with a particular focus on DBPs present in chlorinated drinking water, concentrating on three primary DBP categories (aliphatic, alicyclic, and aromatic). Additionally, it explores pivotal factors influencing DBP formation, encompassing disinfectant types, water source characteristics, and environmental conditions, such as the presence of natural materials in water. The main objective is to discern the most hazardous DBPs, considering criteria such as regulation standards, potential health impacts, and chemical diversity. It provides a catalog of 63 key DBPs alongside their corresponding parameters. From this set, 28 compounds are meticulously chosen for in-depth analysis based on the above criteria. The findings strive to guide the advancement of water treatment technologies and intelligent sensory systems for the efficient water quality surveillance. This, in turn, enables reliable DBP detection within water distribution networks. By enriching the understanding of DBP-associated health hazards and offering valuable insights, this research is aimed to contribute to influencing policy-making in regulations and treatment strategies, thereby protecting public health and improving safety related to chlorinated drinking water quality.

## Abstract 2

To ensure safe and clean drinking water for all, we uncover 63 key disinfection byproducts and provide insights about their formation, regulations, and related health hazards.

## 1 Introduction

Disinfection in water is a vital process to mitigate water-borne diseases (such as cholera and typhoid^1^) and to supply safe drinking water to the public. However, the use of disinfectants in the water produces hundreds or thousands of various disinfection byproducts (DBPs) in the water due to their reaction with natural organic matter (NOM).^2−4^ The formation of DBPs (type and quantity) is related to the disinfectant characteristics (amount of dose and contact time of the disinfectant with the water), as well as the water source characteristics (potential of hydrogen, temperature, NOM, microcontaminants, and inorganic ions).^5^ Moreover, it has been observed that factors such as climate change (i.e., temperature rise) and increasing human population (higher needs for purified drinking water) have promoted DBPs’ formation at high rates.^6,7^

Evidence suggests that increased exposure to DBPs is hazardous to human health as they exhibit high cytotoxicity, mutagenicity, and carcinogenicity; long-term exposure is related to the frequent incidence of carcinogenic, reproductive, and developmental effects.^8,9^

**Figure 1:**
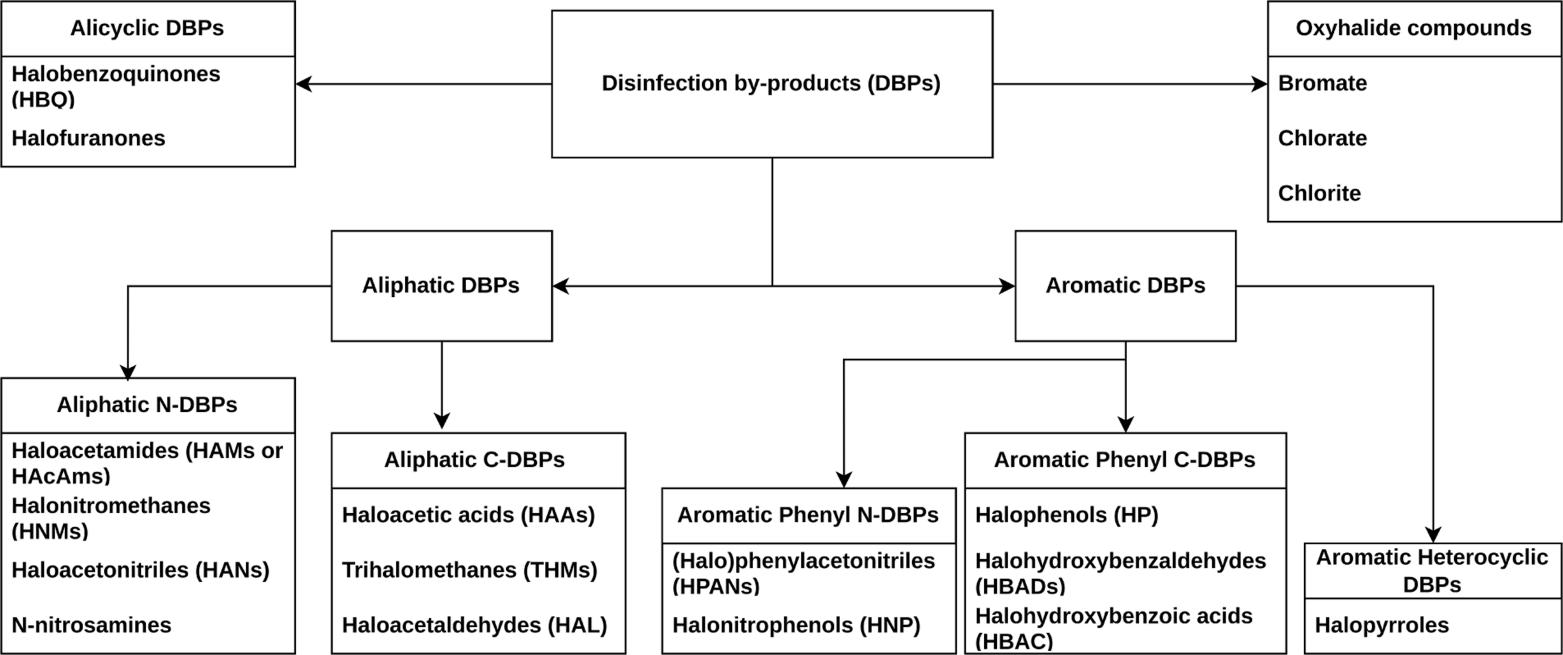
Categories of the main DBPs.

Based on their chemical composition and characteristics, DBPs are divided into three main categories: aliphatic, alicyclic, and aromatic DBPs,^10^ as illustrated in Figure 1. Trihalomethanes (THMs) are a family of aliphatic DBPs that were identified (in 1974) as the first DBP family to be detected.^11^ Since then, over 700 DBPs have been reported in drinking water treatment.^12^ Along with the volatile THMs, the nonvolatile haloacetic acids (HAAs) are considered another major family of DBPs (occurring at a high level in chlorinated drinking water, about 25% of the total DBPs).^13^ Furthermore, the aromatic DBPs (with planar cyclic structures that follow Hückel’s rule) such as halogenated phenols as well as halogenated alicyclic furanone have been identified in chlorinated water.^10,14^ The investigation and identification of these (aromatic and alicyclic) categories are important, as they have higher toxicity than commonly known aliphatic DBPs. However, they are highly unstable as they may degrade into THMs and HAAs.^15^

Despite significant research efforts in the field of DBPs,^16−23^ the understanding of their formation in chlorinated drinking water remains incomplete. It has been observed that the formation of DBPs is affected by certain environmental parameters such as the interaction with various types of NOM, temperature, and pH. However, most of the recent investigations have only considered a specific range of these parameters, such as a temperature range of 20–25 °C and a pH range of 6–8. Moreover, a majority of the available literature reports focus on only a few families of DBPs, while other toxic DBP families, such as aromatic DBPs, are often overlooked. This highlights the need for a comprehensive investigation that considers the formation of DBPs under different scenarios and conditions, including the impact of various types of NOM and water sources together with temperature conditions. A primary concern of the scientific community is the impact of DBPs on human health. Adding information toward filling this research gap can result in the development of more efficient treatment strategies for chlorinated drinking water, which can reduce the formation of harmful DBPs and ensure the delivery of safe drinking water to all individuals.

During 2021, two relevant review papers were published,^13,24^ addressing the formation of DBPs in chlorinated drinking water. Those papers provide a general overview of DBPs, covering aspects such as their occurrence, influencing factors, toxicity, and regulations. In contrast, the current study aims to build upon this existing literature by delving into more specific details and correlations for each DBP compound, utilizing up-to-date information about influencing factors, toxicity, and health implications. The current work also proposes another classification and taxonomy of DBPs, which improves some inconsistencies appearing in ref (13) and in earlier works, from a chemical viewpoint (for example, halofuranones should belong to alicyclic and not aromatic compounds). This work aims to contribute to the existing body of knowledge on DBPs by providing a list of relevant publications, incorporating the latest findings and insights available as of the present date. Moreover, this study analyzes all DBPs, selecting the most critical ones based on their significance and occurrence frequency. This analysis is expected to guide future research efforts in detecting and cleaning DBPs using various treatment techniques.

The paper is organized as follows: Section 2 explains the methodology of the literature survey undertaken to gather information on DBPs. Section 3 provides a brief overview of the three main classes of DBPs (aliphatic, alicyclic, and aromatic). Then, Section 4 provides a detailed explanation of the critical parameters that should be considered in evaluating DBPs. Section 5 covers the impact of DBPs on human health, providing an overview of the known health risks associated with exposure to certain DBPs. Next, Section 6 discusses and lists the most important DBP compounds that need to be prioritized in future studies. Finally, Section 7 outlines the environmental implications of this paper.

## 2 Methodology

The bibliographic analysis consisted of two main steps: (a) collection of relevant literature and (b) detailed review and analysis of the retrieved literature. The following objectives were considered:

1 To perform a comprehensive investigation of DBPs, identifying the most prevalent and toxic ones based on their availability and popularity in chlorinated drinking water, and their impact on human health;
2 To explore the impact of various crucial parameters (such as type of NOM and water source) affecting the formation of DBPs;
3 To identify the existing regulations on DBP used both at the US and the EU levels, identifying existing gaps.

The first step involved a keyword-based search using the popular search engines “IEEE Xplore” and “Elsevier ScienceDirect”, as well as the scientific indexing services “Clarivate Web of Science” and “CAS SciFinder”. The search queries used were: (“disinfection byproducts” and “chlorinated drinking water”) and (“impact of disinfection byproducts” and “human health”). The results were filtered based on relevance and publication date, mainly ranging from 2004 to 2023, with an emphasis on the most recent publications. A few earlier publications (before 2004) were also considered, which provided some useful insights. Initially, several hundred research papers were identified. Subsequently, this number was reduced after eliminating papers with overlapping information, mostly related to DBPs’ general overview, resulting in a total of about a hundred papers for the analysis.

In the second step, collected literature was subjected to in-depth analysis, involving an elaborate examination of the content of the papers retrieved to extract relevant information. The analysis was conducted by identifying the main classes of DBPs reported in each paper and the important parameters that affect their formation. The papers were then grouped based on their findings related to the different classes of DBPs, namely, aliphatic, alicyclic, and aromatic DBPs. For each class, the papers were analyzed for their reported DBP compounds, concentration in water, and potential human health risks. In total, the literature identified 120 DBP compounds. From those, 63 compounds were selected based on their prevalence, accessibility, and potential risks to human health. Compounds with very little accompanying information or little evidence of their occurrence were omitted. Furthermore, the important parameters affecting the formation of DBPs were identified and discussed in more detail. These parameters included: (a) type of water source (surface water or groundwater); (b) name of the disinfectant; (c) types of DBP compounds; (d) measured concentration of DBP compounds in water, as reported in the bibliography; (e) their interaction with NOM; (f) their temperature; and (g) and their response to water’s pH. Regarding the impact of DBPs on human health, the various risks associated with long-term exposure to certain DBPs were recorded. The analysis concluded with the creation of a list of the most important DBP compounds (28 compounds were selected representing the majority of the DBP families), which we propose as the ones that need to be considered in future investigation.

## 3 Overview of DBP Categories

As stated before, the DBPs can be categorized into three distinct categories based on their chemical composition and characteristics. A brief overview of all three categories is expressed in the following subsections. Figure 1 provides a graphical representation of the main categories of DBPs. Moreover, a comprehensive exploration (in Section S2) of these categories and their compounds, along with figures (Figures S1–S16) can be found in the Supporting Information (the notation ‘S’ denotes that the relevant information and figures are referenced in the Supporting Information).

### 3.1 Aliphatic DBPs

Aliphatic DBPs are prevalent in water with two main categories: aliphatic nitrogenous DBPs (aliphatic N-DBPs) and aliphatic carbonaceous DBPs (aliphatic C-DBPs). Aliphatic C-DBPs are more abundant, while aliphatic N-DBPs are more toxic, forming in water with high dissolved organic nitrogen (DON), especially when influenced by wastewater or algae.^25,26^ Water acidity (pH < 6) promotes aliphatic N-DBP formation, contrasting with alkaline conditions (pH > 8) favoring aliphatic C-DBPs.^27^ Major aliphatic N-DBPs are mostly unregulated haloacetamides, halonitromethanes, haloacetonitriles (HANs), and N-nitrosamines. Conversely, commonly studied and regulated aliphatic C-DBPs include THM, HAA, and haloacetaldehydes (more details are available in Section S2.1).

### 3.2 Alicyclic DBPs

While aliphatic and aromatic DBP categories receive more attention, alicyclic DBPs have been less explored. Despite this, investigating alicyclic DBP families, such as halobenzoquinones (HBQ) and halofuranones, is crucial, as they are considered emerging DBPs. Limited resources have focused on these two alicyclic DBP families, and Section S2.2 (in the Supporting Information) offers a summary of their characteristics.

### 3.3 Aromatic DBPs

The final DBP category is aromatic DBPs, characterized by a planar cyclic structure following Hückel’s rule and occurring at lower concentrations than aliphatic DBPs.^10^ Investigating these DBPs is crucial, as popular DBP regulations may not fully mitigate health risks from disinfectant drinking water.^18^ Aromatic DBPs can also act as precursors for aliphatic DBPs.^10^ This category is subdivided into three classes: phenyl nitrogenous DBPs (phenyl N-DBPs), phenyl carbonaceous DBPs (phenyl C-DBPs), and heterocyclic DBPs. Major families include halophenylacetonitriles (HPANs) and halonitrophenols (HNP) for phenyl N-DBPs, and halophenols (HP), halohydroxybenzaldehydes (HBADs), and halohydroxybenzoic acids (HBAC) for phenyl C-DBPs. Halopyrroles are significant among the heterocyclic DBPs. Section S2.3 provides an overview of all of the aromatic DBPs.

### 3.4 Oxyhalide Compounds

In addition to the previously discussed compounds, oxyhalide compounds warrant examination for their impact on human health. These inorganic compounds, containing both oxygen and halogen atoms, include examples such as bromate, chlorate, and chlorite.^28^Section S2.4 offers a concise overview of this category.

## 4 Overview of DBP Formation

As mentioned before, the formation of DBPs is a complicated process that is impacted by several parameters (in different environments) such as the type and dose of disinfectant, the source of the water, water temperature, pH, and the composition of NOM. These parameters are described in more detail in the following subsections.

### 4.1 Type and Dose of Disinfectant

Disinfectants are physical or chemical agents employed in a drinking water supply to eliminate or prevent pathogenic microorganisms from growing. Water disinfection is the method of adding a disinfectant to water and is considered one of the most useful and efficient treatments for preventing water-borne diseases in human life.^29^ The current study focuses specifically on chemical disinfectants. Chlorine (Cl_2_) is the most commonly used chemical disinfectant in water treatment, and the process is known as chlorination of drinking water. It is important to note that the formation of DBPs is directly linked to the type, amount, and reaction rate of the disinfectant used in water treatment. For instance, the reaction pathways of chlorine are contingent upon its reaction rate, determining whether it predominantly reacts with NOM or inorganic compounds, such as bromide and iodide ions. This, in turn, leads to the formation of either THMs and HAAs or brominated THMs and HANs. Furthermore, escalating the disinfectant dose has been shown to correspond to an increased production of DBPs (such as THMs and HAAs).^30^ However, using lower doses of disinfectant may not effectively kill all of the bacteria and viruses in the water, potentially leading to outbreaks of waterborne illnesses. Therefore, it is important to achieve a balance between disinfection efficacy and risk of DBP formation when choosing a disinfectant, thus wisely determining the appropriate dose. To ensure safe treatment of drinking water, standard governing organizations, such as the US-EPA, set standards for acceptable levels of popular disinfectants. This helps to balance the need for effective disinfection with the potential for harmful DBP formation. Physical methods for water disinfection involve using heat, (ultra)sound, or different types of radiation like microwaves, UV-light, or gamma-rays. However, these treatment methods are not the focus of this study. Instead, the study relates to commonly used chemical disinfectants such as chlorine (Cl_2_), chlorine dioxide (ClO_2_), and chloramine (NH_2_Cl). A list of these chemical disinfectants and their maximum contaminant level (MCL) in milligrams per liter (mg/L) for drinking water treatment is provided in Table 1. The factors that influence the formation of DBPs (mainly in chlorinated water) are discussed in the following subsections in more detail.

**Table 1:** Name of the Chemical Disinfectant and Its Maximum Contaminant Level (MCL) Recommended by US-EPA for the Treatment of Drinking Water^31^

name	MCL
chlorine (Cl_2_)	4 mg/L
chlorine dioxide (ClO_2_)	0.8 mg/L
chloramine (NH_2_Cl)	4 mg/L

### 4.2 Source of the Water

Water is essential for human survival and is one of the most important resources on Earth. It is collected from two main sources: the surface and the ground. Surface water is found on the surface of the Earth and is available in streams, rivers, lakes, reservoirs, and oceans. It is considered the most common source of water for drinking and requires proper treatment before it can be consumed. The reason for this is that surface water contains a high concentration of NOM and is vulnerable to contamination by harmful bacteria, parasites, viruses, and other contaminants.^32^ On the other hand, groundwater is found in the saturated zones beneath the land surface and contains low NOM concentrations.^32^ The low concentration of NOM in groundwater makes it less vulnerable to contamination and less likely to form DBPs compared to surface water. Despite this, the amount of groundwater available for consumption is limited and is decreasing day by day due to overpopulation and climate change. Hence, it is critical to ensure the appropriate treatment of surface water to secure the water supply for human consumption. Apart from the source of water, several other factors can affect the formation of DBPs. For example, factors such as rural versus urban areas, seasonal, and climate variations can also impact the formation of DBPs in water. Different situations/conditions affecting the presence of DBPs in various water sources are outlined below.

1 Surface water versus groundwater: surface water usually contains higher levels of NOMs, such as algae and bacteria, which can react with disinfectants to form DBPs. Groundwater, conversely, has lower levels of NOMs and may require less disinfectant. The higher or lower levels of NOM are proportional to the amount of formed DBPs. Therefore, the type and concentration of DBPs formed can differ between these sources of water.^33−39^
2 Rural versus urban areas: in rural water sources, NOM levels arise from agricultural runoff, fertilizer and pesticide applications, animal husbandry, and inefficient irrigation. Conversely, urban water sources exhibit distinct organic matter compositions influenced by industrial effluents, amount of heavy metals, oil, and grease.^40^ The quantifiable impact of these diverse NOMs on water quality is evident in their variable responses to disinfectants, leading to significant shifts in pH and alkalinity. Furthermore, the interactive dynamics of these constituents contribute to the formation of diverse DBPs.
3 Seasonal variations: the concentration of NOM in water varies depending on the season.^41^ During heavy rainfalls, the water runoff increases the amount of NOM in the surface water, and higher doses of disinfectant are required to achieve the desired level of water treatment, which results in higher levels of DBPs.
4 Climate variations: extreme climatic events, like storms and floods, can disrupt water treatment, increasing DBPs. Moreover, changes in water temperature affect the formation of DBPs. During the warmer months, higher temperatures increase the rate of reactions between NOM and disinfectants, resulting in more DBPs being formed.^42−44^

### 4.3 Water Temperature

The temperature of the water can affect the formation of DBPs in several ways. Warm water can increase the formation of DBPs, as microorganisms can grow and multiply more quickly, leading to higher NOM levels.^30^ Additionally, warm water can also increase the rate of chemical reactions, leading to the formation of more DBPs. Conversely, boiled water (heating time at least 5 min) may degrade the formation of DBPs.^45^ Thus, careful consideration of the water temperature is essential in water treatment processes to mitigate the formation of harmful DBPs.

It is also important to consider the impact of the water temperature in combination with the disinfectant used. Research has shown that higher disinfectant doses are required during summer to maintain a sufficient level of residual disinfectant in distribution systems.^46^ This is because warm water can cause the disinfectant residuals to deplete more quickly, making it challenging to maintain a minimal level of residual disinfectant. Furthermore, microbial activity in distribution networks is higher in warm waters than in cold waters.^46,47^ Therefore, in the summer, higher disinfectant doses are used to maintain a sufficient level of residual disinfectant in the distribution system.^46^Table 2 lists the suggested amount of chlorine (as an example of disinfectant) required for different water temperatures, for more effective use,^46^ together with the resulting pH of the water.

**Table 2:** Variation of the Required Amount of Chlorine Doses Corresponding to the Water Temperature and pH^46^

water temperature in °C	pH	amount of Cl in mg/L
4	6.0	0.1 (Blaser et al.^48^)
5	6.0	0.5 (Berman and Hoff^49^)
5	7.0	0.5 (Berman et al.^50^)
5	7.1	0.61 (Rice et al.^51^)
15	6.0	1.5 (Clark et al.^52^)
20	7.0	2 (Clark et al.^52^)

It is worth mentioning that climate variation can also affect the temperature of water sources, leading to changes in the formation of DBPs. For example, in areas that experience more frequent heatwaves, the temperature of water sources may increase, resulting in higher concentrations of DBPs. Finally, it is also worth noting that most of the studies in the literature have reported water temperatures between 20 and 22 °C.

### 4.4 Potential of Hydrogen (pH)

The pH is a critical factor in the formation of DBPs.^13,23,53−58^ The pH level of water represents the acidic (pH < 7.0) or basic/alkaline (pH > 7.0) nature of the water. The pH level of the water affects the solubility and reactivity of different chemicals and substances including NOM and disinfectants. If the pH level is too low or too high, then this can increase the formation of harmful DBPs. For example, at lower pH levels (5.0–6.0), the formation of some HAAs is favored, while at higher pH levels (9.0–10.0), the formation of THMs and nitrogenous DBPs, such as nitrosamines and HANs, is favored.^30^ In addition, the pH level of water can also affect the reactivity of the disinfectants. For instance, at lower pH levels, chlorine is more reactive and can form more DBPs, whereas, at higher pH levels, chlorine is less reactive and the formation of chlorinated DBPs is reduced. Therefore, it is important to maintain a neutral pH level (around 7.0) in water treatment processes to minimize the formation of harmful DBPs. This can be achieved through the pH adjustment and control during the water treatment process. The majority of the relevant literature recorded has followed the standard pH level between 6.5 and 8.5.

### 4.5 Composition of Natural Organic Matter

NOM is a complex organic and slightly water-soluble component that is available in surface water and groundwater. Plant and animal material and waste (dead plants/plant waste such as leaves/bush and tree trimmings/animal manure), wastewater effluents, algal extracellular organic matter, humic and fulvic acids, and free amino acids are mainly available as NOM in water.^59,60^ During disinfection, the disinfectant reacts with the NOM, resulting in the formation of DBPs.^13,34,38,61−63^ However, it is impossible to recognize the specific organic substances (components of the NOM responsible for DBPs) accountable for specific DBPs.^10^ The NOM concentration in surface water and groundwater varies between 2 and 10 mg/L.^64^ Although the amount and composition of NOM in water can vary depending on the source, high levels of NOM can increase the formation of DBPs. Additionally, NOM can also impact the pH levels in water, further influencing the formation of DBPs. Thus, it is important to consider the presence of NOM in water when evaluating the potential for DBP formation and developing appropriate water treatment strategies to minimize DBP formation. Examples of NOM components that can be found in water sources are listed below.

1 Humic substances: humic substances is a class of complex, naturally occurring organic compounds that are formed from the decomposition of plant and animal matter. They are commonly found in water environments such as rivers and lakes and are known to contribute to the color/taste/odor of drinking water.
2 Fulvic acids: fulvic acids are a subclass of humic substances that are characterized by their low molecular weight and high degree of aromaticity. They are commonly found in water environments and are known to contribute to the color/taste/odor of drinking water.
3 Tannins: tannins are a class of polyphenolic compounds that are commonly found in water environments as a result of leaching from leaves and bark of trees and other vegetation.
4 Lignin: lignins are complex polyphenolic compounds that are found in woody plants and are responsible for the mechanical strength and rigidity of plant cell walls. Lignin is commonly found in water environments as a result of the decomposition of plant matter.
5 Polycyclic aromatic hydrocarbons (PAHs): PAHs are a class of organic compounds that contain multiple aromatic rings. They are formed from incomplete combustion of organic matter and are commonly found in water environments as a result of human activities such as industrial processes and transportation.
6 Heterocyclic aromatic compounds (HACs): HACs are a class of organic compounds that contain a heteroatom (typically, nitrogen) inserted in an aromatic ring. Examples include pyrazines, pyridines, and quinolines, which are commonly found in water environments as a result of human activities such as agricultural runoff, industrial processes, and wastewater discharge.
7 Phenols: phenols are a class of organic compounds that contain a hydroxyl group (OH) attached to an aromatic ring (phenyl group). Examples of naturally occurring phenols include catechol, vanillin, and salicylic acid.
8 Aliphatic acids, alcohols, and amines: aliphatic acids (such as acetic and formic acid), aliphatic alcohols (such as methanol and ethanol), and aliphatic amines (such as methylamine and ethylamine) are simple organic compounds that are commonly found in water sources. Aliphatic acids form aliphatic DBPs such as HAAs.

## 5 Potential Impact of DBPs on Human Health

The potential impact of DBPs on human health is a topic of great concern and has been the subject of extensive research. To assess the toxicity level of each DBP, researchers have utilized various methods such as in vivo and in vitro bioassays as well as epidemiologic studies and quantitative structure–activity relationship techniques.^10^ The findings from these studies indicate that exposure to DBPs through ingestion, dermal contact, and inhalation can result in significant health risks, including genotoxicity and cytotoxicity.^21,29,33−36,44,55,65−76^ Genotoxicity refers to the ability of a substance to damage DNA, which can result in mutations and increase the risk of cancer. Cytotoxicity, on the other hand, refers to the ability of a substance to harm or kill cells, which can lead to tissue damage and organ failure. The main potential impacts of DBPs on human health, as discussed in the majority of the research work under study,^46^ are listed below.

1 Carcinogenic effects: long-term exposure to some DBPs, such as THMs and HAAs, has been linked to an increased risk of certain types of cancers, including bladder, liver, and colon cancers.^12,21,29,65,66,77−83^
2 Reproductive and developmental effects: studies have suggested that exposure to high levels of THMs during pregnancy may increase the risk of spontaneous abortion, low birth weight, and neural tube defects in newborns.^29,83−89^
3 Cardiovascular diseases: some DBPs, such as THMs and HAAs, have been associated with an increased risk of cardiovascular disease, including stroke.
4 Respiratory problems: exposure to high levels of DBPs, particularly chloramines, has been linked to respiratory problems, including asthma and bronchitis.^66,78−82^
5 Immune system effects: long-term exposure to DBPs has been shown to weaken the immune system, making individuals more susceptible to infections and illnesses.

## 6 Discussion

This work provides a comprehensive overview of the different classes of DBPs and their compounds, important parameters affecting their formation, and potential human health risks associated with their presence in drinking water. This paper serves a dual role: (a) to create awareness among the general public and policy-makers regarding the harmful effects of DBPs on human health and the importance of regulating their presence in drinking water and (b) to encourage further research on the formation of different categories of DBPs and their impact on human health for the development of improved and updated regulations.

### 6.1 Regulation

In light of the findings regarding the health hazards of DBPs (see Section 5), several organizations, including the World Health Organization (WHO), the United States Environmental Protection Agency (US-EPA), and the European Union (EU), have established regulations for safe drinking water based on evidence of negative human health impacts from DBPs.^9,12,13,29,72,78,83,90^ There are four primary reasons to follow the regulations provided by these organizations:

1 Protect public health: these organizations provide guidelines and regulations to ensure that drinking water is safe for consumption and free from harmful contaminants. Following these regulations can help protect public health and reduce the risk of waterborne illnesses.
2 Legal compliance: in many countries, drinking water systems are required by law to follow the regulations provided by these organizations. Failure to comply with these regulations can result in fines, legal action, and loss of public confidence.
3 International standards: the regulations provided by these organizations are recognized internationally and are widely used by governments and water suppliers around the world. By following these standards, the water provided using the regulated drinking water systems can be ensured to meet international standards and can be trusted by consumers.
4 Environmental protection: the regulations provided by these organizations also consider the impact of water treatment and distribution on the environment. By following these guidelines, drinking water systems can help protect the environment and ensure the sustainability of our water resources.

### 6.2 Analytical Info on DBPs

In light of the significant impact of various categories of DBPs and their compounds on human health, two comprehensive tables (Tables 3 and 4) are provided to offer a concise overview. Table 3 plays a crucial role in elucidating DBPs by systematically presenting key information across various columns. This table is an essential reference for researchers, policymakers, and practitioners seeking a deep understanding of DBPs and their implications. Specifically, the family name (and compound name) column provides vital nomenclature and categorization, facilitating quick identification and classification of DBPs. For instance, the compound name offers nomenclature precision, while the family name categorizes compounds based on shared chemical structures. Additionally, the probable NOM column illuminates the organic matter sources responsible for DBP formation, aiding in the environmental assessment. The column favorable parameters/scenarios lists the favorable scenarios in the existing literature for the formation of DBPs of certain families. Currently, it encompasses factors such as DON, acidic water conditions (pH < 6), intricate ozonation-chlorination sequences, and chloramination scenarios without free chlorine predisinfection. Elevated pH (pH > 8) and temperature, along with the presence of DOC, are recognized as conducive conditions for certain DBPs. Additionally, the column underscores highly oxygen-containing compounds in water. By enhancing the favorable parameters/scenarios column and overall completeness, Table 3 can significantly amplify its impact as an invaluable tool for comprehending DBPs and their categorization, thereby facilitating informed decision-making in the realms of water treatment and safety.

**Table 3:** Characteristics of DBPs in Relation to Family, Category, Probable NOM, and Favorable Parameters/Scenarios^a^^,^^b^

**family name;** compound names	**category**	**probable NOM**	**favorable parameters; scenarios**
**HAMs (HAcAms):** (i) DBAcAm, (ii) DCAcAm, (iii) TCAcAm	aliphatic N-DBPs	DON such as amino acids, pyrroles, and pyrimidines	1. Dissolved organic nitrogen (DON); 2. acidic water (pH < 6)
**HNMs:** (i) TCNM, (ii) BNM	aliphatic N-DBPs	DON and mostly the hydrophilic components of NOM	1. DON; 2. acidic water (pH < 6); 3. ozonation-chlorination, followed by chlorination, ozonation-chloramination, and chloramination.
**HANs:** (i) DCAN, (ii) DBAN, (iii) TCAN	aliphatic N-DBPs	DON such as phenol and resorcinol	1. DON; 2. acidic water (pH < 6)
**NNAs:** (i) NNDMA	aliphatic N-DBPs	reaction of chlorine with organic matter (such as dimethylamine and nitrite)	1. chloramination of compound precursors, particularly without a free chlorine predisinfection
**THM:** (i) CF, (ii) DBCM, (iii) BDCM, (iv) BF	aliphatic C-DBPs	dead plants or plant waste leaves, bush and tree clippings, animal manure	1. higher water pH (pH > 8) and temperature; 2. dissolved organic carbon (DOC)
**HAAs:** (i) DCAA, (ii) TCAA, (iii) MCAA, (iv) MBAA, (v) DBAA	aliphatic C-DBPs	dead plants or plant waste leaves, bush and tree clippings, animal manure	1. higher temperature; 2. DOC
**HAL:** (i) TBAL, (ii) CAL, (iii) DBAL, (iv) BCAL, (v) DBCAL, (vi) IAL, (vii) BAL, (viii) BDCAL, (ix) DCAL, (x) TCAL	aliphatic C-DBPs	organic carbon and acetaldehyde	1. higher water pH (pH > 8) and temperature; 2. DOC
**HBQ:** (i) DCMBQ, (ii) TriCBQ	alicyclic DBPs	lignin-like and highly oxygen-containing components of NOM	1. highly oxygen-containing compounds in the water
**HFur:** (i) TriCMHFur	alicyclic DBPs	NOM with higher aromaticity and heterocyclic structures such as humic substances, fulvic acids, polycyclic aromatic hydrocarbons	NA
**HPANs:** (i) CHPAN, (ii) DCHPAN	aromatic phenyl N-DBPs	NOM with higher aromaticity and more phenyl structures. NOM formed due to the decomposition of plant and animal matter	NA
**HNPs:** (i) DCHNP, (ii) BCHNP, (iii) DBHNP, (iv) DIHNP	aromatic phenyl N-DBPs	NOM with higher aromaticity and more phenyl structures. NOM formed due to the decomposition of plant and animal matter	NA
**HPs:** (i) 2CP, (ii) DCP, (iii) TCP, (iv) 2IP, (v) 4IP, (vi) IMP, (vii) DCBP, (viii) H3IP, (ix) DBCP, (x) TBP, (xi) TIP, (xii) HDIP	aromatic phenyl C-DBPs	NOM with higher aromaticity and more phenyl structures, such as chlorophenol; phenols, lignins, humic acids	NA
**HBADs:** (i) DCHBAD, (ii) BCHBAD, (iii) DBHBAD	aromatic phenyl C-DBPs	NOM with higher aromaticity and more phenyl structures, such as bromophenols; phenols, lignins, humic acids	NA
**HBAC & SAC:** (i) DCHBAC, (ii) DBHBAC, (iii) 3IHBAC, (iv) DIHBAC, (v) DCSAC, (vi) BCSAC, (vii) DBSAC	aromatic phenyl C-DBPs	NOM with higher aromaticity and more phenyl structures, such as bromophenols; phenols, lignins, humic acids	NA
**HPyr:** TBPyr	aromatic heterocyclic DBPs	NOM with higher aromaticity and heterocyclic structures such as humic substances; fulvic acids, polycyclic aromatic hydrocarbons	NA
bromate	oxyhalides	ozonating or chlorinating bromide-containing water	NA
chlorate, chlorite	oxyhalides	generated in hypochlorite solutions	NA
			

a Abbreviations (apply for both Tables 3 and 4).

b NA—not available; DON—dissolved organic nitrogen; DOC—dissolved organic carbon; NOM—natural organic matter; HAMs or HAcAms—haloacetamides; DBAcAm—2,2-dibromoacetamide; DCAcAm—2,2-dichloroacetamide; TCAcAm—2,2,2-trichloroacetamide; HNMs—halonitromethanes; TCNM—trichloronitromethane; BNM—bromonitromethane; HANs—haloacetonitriles; DCAN—2-dichloroacetonitrile; DBAN—2,2-dibromoacetonitrile; TCAN—2,2,2-trichloroacetonitrile; NNAs—nnitrosamines; NNDMA—N-nitrosodimethylamine; For the remaining abbreviations, see the footnote of Table 4.

**Table 4:** Characteristics of DBPs Including the Compound Family, Name, Stability, Regulations, Estimated Concentration, Detection Frequency, and Probable Health Issues^a^,^b^

family name; compound names	stability of the compound	regulations	estimated concentration (family/compound)	detection frequency	probable health issues
**HAMs (HAcAms):** (i) DBAcAm, (ii) DCAcAm, (iii) TCAcAm	generally not stable in water, hydrolyze at higher pH and temperature (*T*)	NA	0–7.4 μg/L (family)	low	cytotoxic to two exposure pathway-related cell lines: (i) human gastric epithelial cell line GES-1 and (ii) immortalized human keratinocyte cell line HaCaT. HAMs are 142.2 times more toxic than aliphatic CDBPs and 1.4 times more toxic than other aliphatic NDBPs
**HNMs:** (i) TCNM, (ii) BNM	generally not stable in water, hydrolyze at higher pH and *T*	NA	0–10 μg/L (family)	low	genotoxic as well as cytotoxic inducing high levels of DNA signature breaks
**HANs:** (i) DCAN	generally not stable in water, hydrolyze at higher pH and *T*	WHO: 20 μg/L; US-EPA: 6 μg/L	3–14 μg/L	high	HANs family (1-3): 1. increase of fetal resorption and reduction in fetal body weight
**HANs:** (ii) DBAN		WHO: 70 μg/L; US-EPA: 20 μg/L	26.6 μg/L	high	2. responsible for cancer, mutagenic and clastogenic effects
**HANs:** (iii) TCAN		NA	3–14 μg/L	medium	3. responsible for the damages of liver and kidney
**NNAs:** (i) NNDMA	generally not stable in water, hydrolyze at higher pH and *T*	WHO: 0.1 μg/L; US-EPA: 0.01 μg/L	10 ng/L	low	extremely genotoxic, cytotoxic, and mutagenic
**THM:** (i) CF	stable in water (THM family)	EU: 100 μg/L; US-EPA: 70 μg/L	4–164 μg/L (family)	high	THM family (1-4): 1. cancer (CF, BDCM, BF)
**THM:** (ii) DBCM		US-EPA: 60 μg/L		high	2. liver and kidney damage (CF, BDCM, DBCM, BF)
**THM:** (iii) BDCM		US-EPA: 45 μg/L		high	3. reproductive effects (CF, BDCM, DBCM)
**THM:** (iv) BF		US-EPA: 6 μg/L		high	4. nervous system damage (DBCM, BF)
**HAAs:** (i) DCAA	stable in water (HAAs family)	US-EPA: 60 μg/L	5–130 μg/L (family)	high	HAAs family (1-4): 1. bladder cancer
**HAAs:** (ii) TCAA		US-EPA: 20 μg/L		high	2. reproductive abnormalities
**HAAs:** (iii) MCAA		US-EPA: 70 μg/L		high	3. liver and kidney damage
**HAAs:** (iv) MBAA, (v) DBAA		US-EPA: 60 μg/L		high	4. developmental consequences
**HALs:** (i) TBAL	info not available	NA	1–25 μg/L	low	highly cytotoxic and genotoxic
**HALs:** (ii) CAL		NA	0.25–10 μg/L	low	(entire HALs family)
**HALs:** (iii) DBAL		NA	0.25–10 μg/L	low	
**HALs:** (iv) BCAL		NA	0.1–10 μg/L	low	
**HALs:** (v) DBCAL		NA	1–25 μg/L	low	
**HALs:** (vi) IAL		NA	0.5–8 μg/L	low	
**HALs:** (vii) BAL		NA	0.5–10 μg/L	low	
**HALs:** (viii) BDCAL		NA	1–25 μg/L	low	
**HALs:** (ix) DCAL		NA	0.25–10 μg/L	low	
**HALs**: (x) TCAL		China 10 μg/L	0.25–10 μg/L	medium	
**HBQ:** (i) DCMBQ, (ii) TriCBQ	unstable in the drinking water	NA	0.5–275 ng/L (family)	low	1. extremely cytotoxic, may be genotoxic and carcinogenic; 2. cellular protein and DNA damage, bladder cancer
**HFur:** (i) TriCMHFur	unstable and they are easily hydrolyzed under alkaline conditions	NA	100 ng/L	low	genotoxicity and mutation effects, potent carcinogenic DBPs that can cause cancer
**HPANs:** (i) CHPAN, (ii) DCHPAN	stable in the drinking water. Yet, chloramine with poor oxidative capacity may result in the formation of aliphatic DBPs	NA	530 ng/L (family)	low	extremely cytotoxic and cause developmental effects
**HNPs:** (i) DCHNP, (ii) BCHNP, (iii) DBHNP, (iv) DIHNP	stable in the drinking water. Yet, chloramine with poor oxidative capacity may result in the formation of aliphatic DBPs	NA	5 ng/L (family)	low	extremely cytotoxic and cause developmental effects
**HPs:** (i) 2CP, (ii) DCP, (iii) TCP, (iv) 2IP, (v) 4IP, (vi) IMP, (vii) DCBP, (viii) H3IP, (ix) DBCP, (x) TBP, (xi) TIP, (xii) HDIP	stable in the drinking water. Yet, chloramine with poor oxidative capacity may result in the formation of aliphatic DBPs	NA	2.5–32 ng/L (family)	low	cytotoxic, causing developmental abnormalities, endocrine disturbance, and growth inhibition problems; cause and promoters of cancer and tumors
**HBADs:** (i) DCHBAD, (ii) BCHBAD, (iii) DBHBAD	stable in the drinking water. Yet, chloramine with poor oxidative capacity may result in the formation of aliphatic DBPs	NA	very low (family)	low	cytotoxic and have developmental consequences; growth inhibitory effects
**HBAC & SAC:** (i) DCHBAC, (ii) DBHBAC, (iii) 3IHBAC, (iv) DIHBAC, (v) DCSAC, (vi) BCSAC, (vii) DBSAC	stable in the drinking water. Yet, chloramine with poor oxidative capacity may result in the formation of aliphatic DBPs	NA	70.2 ng/L (family)	low	cytotoxic and can induce growth inhibition
**HPyr:** TBPyr	unstable and easily hydrolyzed under alkaline conditions	NA	61 μmol/L	low	toxic and have a negative effect on the developmental growth
oxyhalides (bromate)	stable in the drinking water	US-EPA: 10 μg/L	0–19.6 μg/L	high	genotoxic and carcinogenic; risk of cancer
oxyhalides (chlorate, chlorite)	stable in the drinking water	WHO: 700 mg/L; US-EPA: 1000 μg/L	NA	high	can harm the blood cells; mutagenic effect
					

a Abbreviations (apply for both Tables 3 and 4).

b WHO—World Health Organization; US-EPA—United States Environmental Protection Agency; THM—trihalomethanes; CF—chloroform; DBCM—dibromochloromethane; BDCM—bromodichloromethane; BF—bromoform; HAAs—haloacetic acids; DCAA—dichloroacetic acid; TCAA—trichloroacetic acid; MCAA—monochloroacetic acid; MBAA—monobromoacetic acid; DBAA—dibromoacetic acid; HAL—haloacetaldehydes; TBAL—tribromoacetaldehyde; CAL—chloroacetaldehyde; DBAL—dibromoacetaldehyde; BCAL—bromochloroacetaldehyde; DBCAL—dibromochloroacetaldehyde; IAL—iodoacetaldehyde; BAL—bromoacetaldehyde; BDCAL—bromodichloroacetaldehyde; DCAL—dichloroacetaldehyde; TCAL—chloral hydrate (hydrated trichloroacetaldehyde); HBQ—halobenzoquinones; DCMBQ—2,6-dichloro-3-methyl-1,4-benzoquinone; TriCBQ—2,3,6-trichloro-1,4-benzoquinone; HFur—halofuranones; TriCMHFur—trichloro-4-methyl-5-hydroxy-2(5*H*)-furanone; HPANs—halophenylacetonitriles; CHPAN—2-chlorophenylacetonitrile; DCHPAN—2,5-dichlorophenylacetonitrile; HNPs—halonitrophenols; DCHNP—2,6-dichloro-4-nitrophenol; BCHNP—2-bromo-6-chloro-4-nitrophenol; DBHNP—2,6-dibromo-4-nitrophenol; DIHNP—2,6-diiodo-4-nitrophenol; HPs—halophenols; 2CP—2-chlorophenol; DCP—2,4-dichlorophenol; TCP—2,4,6-trichlorophenol; 2IP—2-iodophenol; 4IP—4-iodophenol; IMP—4-iodo-2-methylphenol; DCBP—2,6-dichloro-4-bromophenol; H3IP—4-hydroxy-3-iodophenol; DBCP—2,6-dibromo-4-chlorophenol; TBP—2,4,6-tribromophenol; TIP—2,4,6-triiodophenol; HDIP—4-hydroxy-3,5-diiodophenol; HBADs—halohydroxybenzaldehydes; DCHBAD—3,5-dichloro-4-hydroxybenzaldehyde; BCHBAD—3-bromo-5-chloro-4-hydroxybenzaldehyde; DBHBAD—3,5-dibromo-4-hydroxybenzaldehyde; HBAC—halohydroxybenzoic acids; DCHBAC—3,5-dichloro-4-hydroxybenzoic acid; DBHBAC—3,5-dibromo-4-hydroxybenzoic acid; 3IHBAC—3-iodo-4-hydroxybenzoic acid; DIHBAC—3,5-diiodo-4-hydroxybenzoic acid; SAC—salicylic acid; DCSAC—3,5-dichlorosalicylic acid; BCSAC—3-bromo-5-chlorosalicylic acid; DBSAC—3,5-dibromosalicylic acid; HPyr—halopyrroles; and TBPyr—2,3,5-tribromopyrrole.

Many researchers have attempted to formally correlate environmentally favorable parameters with the formation of DBPs, aiming to predict the existence of DBPs based on the environmental conditions within chlorinated drinking water or water distribution systems. A review paper published in 2004 identifies and presents 29 predictive models that have been developed based on data generated in laboratory-scaled and field-scaled investigations,^46^ correlating various DBPs with a range of environmental parameters. Their objective was to review DBP predictive models, identify their advantages and limitations, and examine their potential applications as decision-making tools for water treatment analysis, epidemiological studies, and regulatory concerns. Another review paper, published in 2009, documented more than 48 scientific publications reporting 118 models for predicting DBP formation in drinking water.^91^ The primary areas of focus were THM formation followed by HAAs. Both regression and kinetic models have been proposed in the literature, some of which are mentioned in the current work. In Absalan et al.,^92^ chlorine and THMs were predicted using a variable rate model developed by Hua.^93^ This model successfully predicted THM levels at 86% of the study points. In Rodriguez et al.,^94^ a regression-based model for THM formation was proposed, applicable to both bench-scale conditions and field-scale studies. Additionally, Amy et al.^95^ introduced linear and nonlinear regression models for predicting total THM formation potential and kinetics during the chlorination of natural water. In general, most published models predicting THM formation are based on data generated from chlorination experiments conducted with very high chlorine doses, which are not comparable to the real doses applied in water treatment. The work by Sérodes et al.^41^ identified seasonal variations in THMs and HAAs, primarily associated with variations in organic precursors and changes in water temperature, with the highest occurrence of DBPs observed in spring.

On the other hand, many studies indicate that the one- and two-carbon-atom DBPs currently under scrutiny contribute only approximately 16% to the cytotoxicity of disinfected water,^96^ there is a pressing need to pinpoint the drivers of toxicity within the less understood higher-molecular-weight DBP fraction (beyond two carbon atoms). This study recognizes the importance of delving into the discussion on high-molecular-weight DBPs, as highlighted in a recent review.^97^ The review provides a comprehensive overview of the current understanding of this DBP fraction, which, until recently, has received limited attention. Mitch et al. present innovative analytical approaches for characterizing the diverse structures within this higher-molecular-weight DBP category.^97^

Returning to the details presented in Table 4, it furnishes additional information about the stability of each compound, legal regulations, or guidelines regarding their maximum recommended concentration in drinking water, the estimated concentration range of each compound and its family, detection frequency in water sources, and probable health issues that may arise from exposure to each compound. The stability of the compound column offers crucial insights into the stability profiles of DBPs, specifically in response to pH and temperature variations. The column content reveals diverse stability characteristics, indicating that certain DBPs are generally not stable in water as they tend to hydrolyze at higher pH and temperature. Conversely, some DBPs exhibit stability in water, showcasing resistance to hydrolysis. However, for some stable DBPs, the presence of chloramine with poor oxidative capacity may lead to the formation of aliphatic DBPs. Moreover, a noteworthy portion lacks available information, emphasizing the need for further research.

The regulations column assumes a pivotal role in recognizing the presence of legal guidelines (of diverse regulatory standards), underscoring their significance in ensuring the safety of drinking water and safeguarding public health. Entries include instances of adherence to WHO and US-EPA limits, emphasizing permissible concentrations for specific compounds. Additionally, entries reflect regulations set by the EU and China, showcasing regional variations in standards. The review underscores the importance of adhering to national and international guidelines, with specific attention to compound families, thus emphasizing the multifaceted nature of regulatory frameworks that contribute to the maintenance of safe drinking water. Entries labeled as “NA” indicate areas where specific regulatory information is not available, highlighting the need for further research or clarity in these domains.

The estimated concentration (family/compound) column serves as a crucial repository of numerical values, encapsulating the estimated concentration ranges (identified by researchers) for DBP families/compounds. This information provides invaluable insights into the prevalence of DBPs within water sources, facilitating a nuanced understanding of their potential risks. The column presents a spectrum of concentration ranges (in μg/L), offering a quantitative lens of the abundance of DBPs. Additionally, entries indicating “very low” concentrations and instances marked as “NA” highlight negligible levels or areas lacking specific concentration information. This comprehensive numerical representation equips researchers with quantitative data, empowering them to assess the distribution and potential impact of DBPs in water sources.

The detection frequency column reveals how frequently DBPs are identified in water sources (by researchers) and provides insights into their occurrence patterns. The column content spans varying detection frequencies, ranging from “low” to “high” which illuminates the prevalence of DBPs. Notably, high detection frequencies carry significance as they may indicate potential exposure risks, prompting the need for further attention and in-depth study.

Finally, the probable health issues column emerges as a critical component, providing crucial insights into the potential health implications associated with each DBP. The column content spans a spectrum of health concerns, offering a detailed overview of the risks posed by these compounds. Noteworthy examples include cytotoxicity, mutagenicity, carcinogenicity, and organ-specific effects, which underscore the diverse health risks linked to DBP exposure. Specific entries detail the compounds’ impact on cellular and DNA damage, potential carcinogenic effects, reproductive abnormalities, liver and kidney damage, developmental consequences, and growth inhibition. For instance, some DBPs are cited as being extremely cytotoxic, genotoxic, and carcinogenic, posing risks such as bladder cancer, reproductive abnormalities, and developmental effects. Others may induce fetal resorption, reduce fetal body weight, and contribute to cancer, mutagenic effects, and clastogenic effects.

In summary, Tables 3 and 4 collectively serve as a comprehensive and invaluable resource encompassing vital scientific data about DBPs. These tables offer critical insights into DBPs, ranging from nomenclature, stability, and regulatory compliance to prevalence, occurrence, and potential health impacts. Their structured presentation equips researchers, policymakers, and practitioners with essential information to facilitate informed decision-making and enhance the safety of drinking water.

### 6.3 Selection of Critical DBPs

The primary objective of this subsection is to establish a systematic prioritization of DBP compounds based on specific criteria, incorporating regulatory standards set by prominent public health organizations (US-EPA, EU, WHO) as well as considering the observed and probable severity of health impacts. Furthermore, a key criterion involves ensuring chemical diversity by including at least one compound from each major DBP family. The resulting filtered list, comprising 28 DBP compounds, is meticulously curated and presented in Table 5, derived from comprehensive data extracted from Tables 3 and 4. This selection process unfolds based on **three key criteria**, as follows:

1 **Regulatory standards**: the initial focus centers on compliance with regulatory standards. All regulated DBPs (serial numbers 1–16) are purposefully chosen based on the regulations information available in Table 4 (column ‘regulations’). It ensures alignment with established guidelines set by authoritative public health organizations. By prioritizing regulated DBPs, the selection inherently emphasizes compounds that are subject to rigorous scrutiny, reflecting their recognized impact on human health and the environment. This criterion serves as a robust foundation, providing a basis for comprehensive understanding and targeted management strategies.
2 **Health impact severity**: the subsequent consideration involves the observed and probable severity of health impacts available in Table 4 (column “probable health issues”), encompassing both regulated and unregulated DBPs (serial number 1–28). This criterion acknowledges that the absence of regulation does not diminish the significance of certain DBPs in terms of health impact. By incorporating this criterion, the selection process strives to capture a comprehensive range of health implications associated with different DBP compounds. It thus enhances the overall relevance and applicability of the curated list in addressing potential health concerns.
3 **Chemical diversity**: the final and critical aspect involves the inclusion of at least one DBP compound from each major DBP family within the unregulated category (serial numbers 17–28). This criterion is paramount as different DBP families exhibit distinct chemical properties, reactivity, and potential health effects. This criterion ensures a representative selection that spans the spectrum of chemical characteristics, enriching the curated list with a comprehensive overview of DBP diversity. By including compounds from various families, the selection process aims to uncover potential correlations between chemical structure and health impact, contributing valuable insights to the understanding of DBP behavior in water systems.

**Table 5:** List of Proposed Critical DBPs Considering the Three Criteria

serial number	name of the compound	name of the DBP family	criteria
1	chloroform (CF)	trihalomethanes (THM)	1, 2, 3
2	dibromochloromethane(DBCM)	trihalomethanes (THM)	1, 2, 3
3	bromodichloromethane (BDCM)	trihalomethanes (THM)	1, 2, 3
4	bromoform (BF)	trihalomethanes (THM)	1, 2, 3
5	dichloroacetic acid (DCAA)	haloacetic acid (HAA)	1, 2, 3
6	trichloroacetic acid (TCAA)	haloacetic acid (HAA)	1, 2, 3
7	monochloroacetic acid (MCAA)	haloacetic acid (HAA)	1, 2, 3
8	monobromoacetic acid (MBAA)	haloacetic acid (HAA)	1, 2, 3
9	dibromoacetic acid (DBAA)	haloacetic acid (HAA)	1, 2, 3
10	2-dichloroacetonitrile (DCAN) (C_2_HCl_2_N)	haloacetonitriles (HANs)	1, 2, 3
11	2,2-dibromoacetonitrile (DBAN) (C_2_HBr_2_N)	haloacetonitriles (HANs)	1, 2, 3
12	bromate	oxyhalide compounds	2, 3
13	chlorate	oxyhalide compounds	2, 3
14	chlorite	oxyhalide compounds	2, 3
15	N-nitrosodimethylamine (NNDMA)	N-nitrosamines (NNAs)	2, 3
16	chloral hydrate (hydrated trichloroacetaldehyde)	haloacetaldehydes (HAL)	1, 2, 3
17	2,2-dichloroacetamide (DCAcAm)	haloacetamides (HAMs or HAcAms)	2, 3
18	2,2-dibromoacetamide (DBAcAm)	haloacetamides (HAMs or HAcAms)	2, 3
19	bromonitromethane (BNM)	halonitromethanes (HNMs)	2, 3
20	trichloronitromethane (TCNM)	halonitromethanes (HNMs)	2, 3
21	2,3,6-trichloro-1,4-benzoquinone (TriCBQ)	halobenzoquinones (HBQ)	2, 3
22	trichloro-4-methyl-5-hydroxy-2(5*H*)-furanones	halofuranones	2, 3
23	2-chlorophenylacetonitrile (CPAN)	(halo)phenylacetonitriles (HPANs)	2, 3
24	2,6-dichloro-4-nitrophenol (2,6-DCNP)	halonitrophenols (HNP)	2, 3
25	2,4,6-trichlorophenol	halophenols (HP)	2, 3
26	3-bromo-5-chloro-4-hydroxy-benzaldehyde	halohydroxybenzaldehydes (HBADs)	2, 3
27	3,5-dichlorosalicylic acid	halohydroxybenzoic acids (HBAC)	2, 3
28	2,3,5-tribromopyrrole (TBPR)	halopyrroles	2, 3

Selecting and prioritizing DBPs based on their occurrence and potential impact on human health is an important effort and exercise, considering the existence of tens or hundreds of DBPs and the need to focus on the most significant ones for practical reasons. Achieving a common agreement and consensus on the most harmful and abundant ones allows for better organization, coordination, and execution of research and innovation initiatives in a more targeted manner, thereby addressing the disinfection problem more effectively or even taking actions to mitigate the possibilities of these DBPs forming. For instance, this could involve better monitoring of the precise environmental parameters that facilitate and lead to the formation of these DBP compounds. Furthermore, this effort is essential from a policy perspective as it helps us understand which DBPs remain unregulated, thereby prioritizing regulation or improving the regulation of existing ones. Finally, reaching an agreement on certain critical DBPs permits the assessment of the effectiveness of various treatment methods and the overall hygiene of existing chlorinated drinking water systems and distribution networks.

## 7 Environmental Implications and Summary

The findings of this study pave the way for diverse avenues of future research. To begin, a key challenge lies in pinpointing the specific type of NOM responsible for generating distinct DBP compounds—a task that has remained an ongoing struggle in related studies.^10^ Thus, an imperative area for exploration involves delving deeper into the effects of different NOM types on various DBPs, particularly when they interact in varying environments. Furthermore, there is a call for more comprehensive methods to investigate emerging DBPs and their associated health implications, as new compounds could surface in the future.^98,99^ Long-term health repercussions due to chronic DBP exposure and the evaluation of treatment methods represent vital directions for prospective research.

Advancements in analytical techniques and sensor technologies have the potential to revolutionize DBP detection and monitoring in real time, facilitating proactive strategies for mitigation. Emphasis on emerging sensor technologies, novel analytical approaches, incorporation of artificial intelligence, and computer vision should drive future developments in this domain.

This paper extensively examines the impact of DBPs on human health, focusing on chlorinated drinking water. It includes a comprehensive analysis of the three primary DBP categories, highlighting the critical factors influencing their formation. Identification of hazardous DBPs, such as THMs, HAAs, and other toxic compounds, enhances the understanding of health risks from DBP exposure.

The exhaustive list of DBP compounds, along with their associated parameters, presented in this study serves as a valuable reference for researchers and industry practitioners. It acts as a cornerstone for future inquiries into the presence and impact of DBPs across diverse water sources, facilitating the creation of targeted treatment technologies and monitoring systems.

Lastly, the investigated literature provides a robust foundation for ongoing research, highlighting the persistent importance of addressing health risks of DBPs in drinking water. Leveraging insights from this study, researchers can contribute to the development of strategies to ensure water safety and public health.

Summing up, the selected findings of our work are as follows:

### 7.1 Achievements

1 Explored literature to understand crucial environmental parameters impacting DBP formation in drinking water.
2 Conducted a comprehensive investigation, identifying prevalent and toxic DBPs in chlorinated drinking water and assessing their impact on human health.
3 Recorded existing DBP regulations at the US and EU levels, identifying gaps.

### 7.2 Conclusions on Environmental Parameters—Link with DBPs

1 pH is crucial for DBP detection and higher temperature accelerates chemical reactions.
2 NOM in water contributes to DBP formation.
3 Dissolved oxygen in water affects the NOM and DON reaction leading to DBP formation.

### 7.3 Conclusions on Regulations

1 Most DBPs are unregulated.
2 Known regulations on selected DBPs are from WHO, US-EPA, and EU.
3 For most DBPs, the estimated concentration range is regulated between 10 and 70 μg/L.

### 7.4 Key Challenges

1 Pinpoint the specific NOM types yielding distinct DBPs.
2 Understand and quantify exposure to DBPs posing substantial health risks.
3 Regulate (at least the most important) DBPs and find consensus among policymakers globally.

## Supporting Information Available

Chemical structures representing distinct families of DBPs, comprehensive exploration and analysis of various DBP categories, chemical structures offering insights into the molecular compositions, and analysis providing a thorough examination of diverse DBP characteristics (PDF)
