# Supplementary material for: Assessing the Health Impact of Disinfection Byproducts in Drinking Water
Source: ACS ES T Water. 2024 Mar 2;4(4):1564–78. doi: 10.1021/acsestwater.3c00664 (PMC11019713; doi:10.1021/acsestwater.3c00664)
Supplement: Supplementary file 1 — ew3c00664_si_001.pdf [file ew3c00664_si_001.pdf]

Supporting Information

Assessing the Health Impact of Disinfection Byproducts in Drinking Water

Indrajit Kalita, *,†,‡ Andreas Kamilaris,‡,¶ Paul Havinga,¶ and Igor Reva, *,§

† Computing & Data Sciences (CDS), Boston University, Boston, Massachusetts 02215, USA

‡ CYENS Centre of Excellence, Nicosia 1016, Cyprus

¶ Pervasive Systems Group, University of Twente, Enschede 7522, Netherlands

§ Department of Chemical Engineering, CERES, University of Coimbra, Coimbra 3030-790, Portugal

*Corresponding authors E-mails:

Indrajit Kalita: indrajit@bu.edu

Igor Reva: reva@eq.uc.pt

https://doi.org/10.1021/acsestwater.3c00664

S1. Chemical structures of individual families of DBPs:

Haloacetamides (HAMs): The general haloacetamide structure is represented in Figure S1. The X is a halogen atom (Cl, Br, I); Y and Z are halogens (Cl, Br, I) or hydrogen atoms.

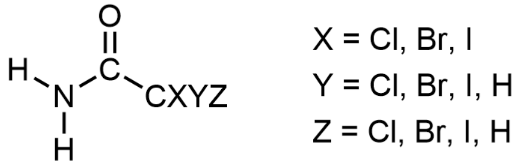

Figure S1: Chemical structure of HAM

Halonitromethanes (HNMs): The chemical structure of HNMs can be seen in Figure S2. It contains both halogen and nitro functional groups and the general chemical structure of an HNM can be represented as $CXYZ-NO_2$, where X is a halogen atom such as chlorine, bromine, or iodine; Y and Z are halogens (Cl, Br, I) or hydrogen atoms, and NO_2 is a nitro group ($-NO_2$) attached to the central carbon atom.

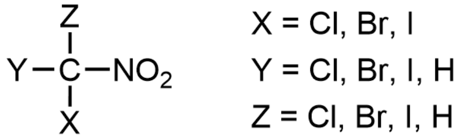

Figure S2: Chemical structure of HNM

Haloacetonitriles (HANs): Haloacetonitriles are a group of organic compounds that contain both halogen and cyano (nitrile) functional groups. The chemical structure of HAN is shown in Figure S3. The structure can be represented as $CXYZ-CN$, where X is a halogen atom such as chlorine, bromine, or iodine; Y and Z are halogens (Cl, Br, I) or hydrogen atoms, and CN is a cyano group ($-C{\equiv}N$) attached to the carbon atom.

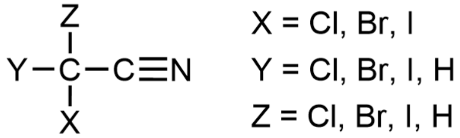

Figure S3: Chemical structure of HAN

N-nitrosamines (NNAs): N-nitrosamines are organic compounds that contain a nitroso group ($-N{=}O$) bonded to an amine group ($-NH_2$). The general chemical structure of an N-nitrosamine can be represented as $R^1-N(-R^2)-N{=}O$, where R^1 and R^2 are organic substituents attached to the nitrogen atom. Figure S4 represents the chemical structure of N-nitrosamine.

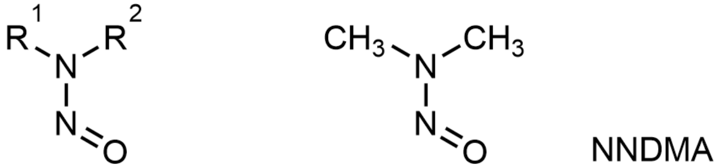

Figure S4: Chemical structure of NNA (left) and NNDMA (right)

Trihalomethanes (THMs): Trihalomethanes are a group of organic compounds that contain three halogen atoms and one carbon atom. Trihalomethanes are a group of organic compounds that contain three halogen atoms and one carbon atom. The general chemical structure of THM is represented in Figure S5. X, Y, and Z represent halogen atoms such as chlorine, bromine, or iodine that can be the same or different.

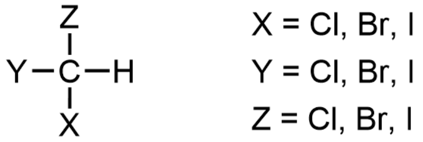

Figure S5: Chemical structure of THMs.

Haloacetic acids (HAAs): Haloacetic acids (HAA) are a group of organic compounds that contain both a halogen and the carboxylic acid functional group. The general chemical structure of HAA can be represented as $CXYZ-COOH$ and is shown in Figure S6. Here, X is a halogen atom (such as chlorine, bromine, or iodine), and Y, Z are halogens (Cl, Br, I) or hydrogen atoms, and $COOH$ is a carboxylic acid group ($-COOH$) attached to the central carbon atom.

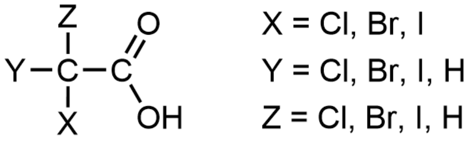

Figure S6: Chemical structure of HAAs.

Haloacetaldehydes (HAL): Haloacetaldehydes (HAL) are organic compounds that consist of a halogen and the aldehyde functional group. The general chemical structure for HAL is shown in Figure S7. X is a halogen atom such as chlorine, bromine, or iodine; Y and Z are halogens (Cl, Br, I) or hydrogen atoms, and −CH(=O) is an aldehyde group linked to the central carbon atom.

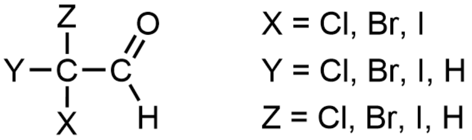

Figure S7: Chemical structure of HALs.

Halobenzoquinones (HBQ): Halobenzoquinones (HBQ) are organic compounds that feature both halogen and benzoquinone moieties. The general chemical structure of HBQ is shown in Figure S8. X, Y, and Z are halogen atoms such as chlorine, bromine, or iodine, and Y can also be an alkane substituent, such as methyl group. The halogen atom can be the same or different.

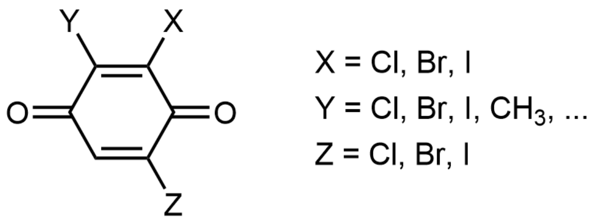

Figure S8: Chemical structure of HBQs.

Halofuranones (HFur): Halofuranones are organic compounds that contain a halogen atom attached to a furanone ring. The furanone ring is a five-membered lactone. The general chemical formula for a halofuranone is $C_4H_3XO_2$, where X represents a halogen atom such as chlorine, bromine, or iodine. Depending on the position of the double C=C bond, the furanone ring can adopt two isomeric forms, *3H* and *5H* (see Figure S9).

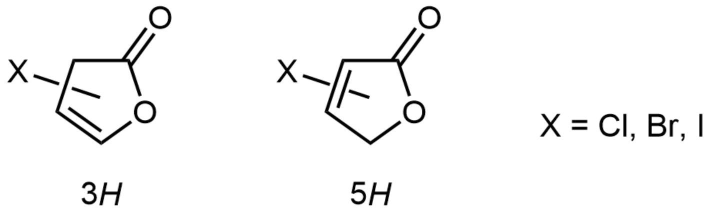

Figure S9: Chemical structure of Halofuranones.

Halophenylacetonitriles (HPANs): The general formula of HPANs is presented in Figure S10. It is important to note that (here and hereafter), substituent X linked to the center of the ring means that the respective group (X in this case) can have variable attachment points at the ring.

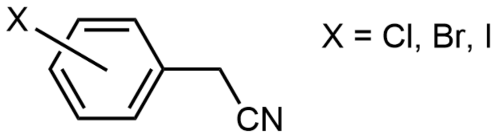

Figure S10: Chemical structure of HPAN.

Halonitrophenols (HNP): Halonitrophenols (HNP) is a class of organic compounds that simultaneously possess halogen (X, Y) and nitro (*NO_2*) functional groups connected to the phenol ring as shown in Figure S11. X and Y are halogen atoms such as chlorine, bromine, or iodine which can be the same or different.

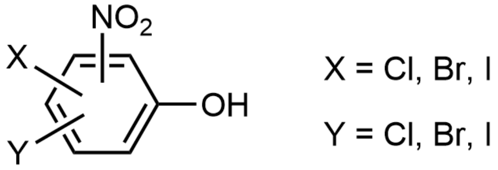

Figure S11: Chemical structure of HNP.

Halophenols (HP): HP are organic compounds that consist of a phenol ring and one or more halogen atoms attached to it. The chemical structure of HP can be found in Figure S12. X, Y, and Z are halogen atoms such as chlorine, bromine, or iodine which can be the same or different; Y and Z can be also hydrogen atoms.

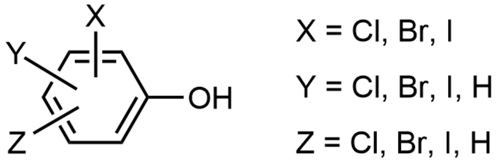

Figure S12: Chemical structure of HP.

Halohydroxybenzaldehydes (HBADs): HBAD is a group of chemical compounds that contain both halogen (chlorine, bromine, or iodine) and hydroxyl functional groups attached to a benzene ring with an aldehyde group (−CHO) at one end. The chemical structure of HBAD is shown in Figure S13.

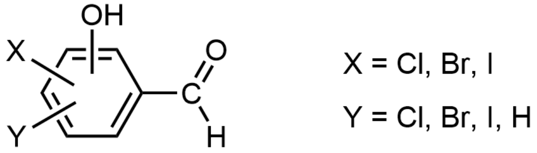

Figure S13: Chemical structure of HBAD.

Halohydroxybenzoic acids (HBAC): HBAC are organic compounds that contain both a halogen atom and a hydroxyl (−OH) group attached to a phenolic ring with a carboxylic acid (−COOH) group. The general chemical formula for a halohydroxybenzoic acid is $C_6H_3X(OH)(COOH)$ or $C_6H_2XY(OH)(COOH)$, where X and Y represent halogen atoms such as chlorine, bromine, or iodine (the same or different), and Y can be also a hydrogen atom. Without any halogen substitution, and with the OH and COOH groups at adjacent (ortho) positions of the ring, this ortho-hydroxybenzoic acid is also called salicylic acid (SAC). The chemical structure of HBAC is depicted in Figure S14, while the structure of salicylic acid is shown in Figure S15.

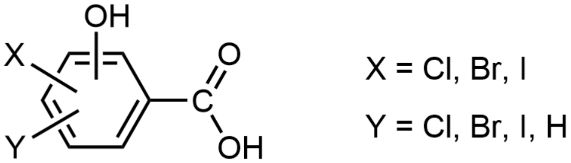

Figure S14: Chemical structure of HBAC.

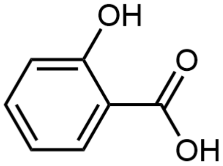

Figure S15: Chemical structure of salicylic acid.

Halopyrroles (HPyr): Halopyrroles are organic compounds that contain a halogen atom attached to the pyrrole ring (a five-membered heterocyclic ring that consists of four carbon atoms and one nitrogen atom). The general chemical formula for a halopyrrole is C_4H_3XNH, where X represents a halogen atom such as chlorine, bromine, or iodine. The structure of the halopyrroles is shown in Figure S16.

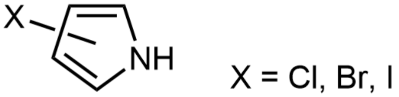

Figure S16: Chemical structure of halopyrrole.

S2. Comprehensive exploration and analysis of diverse DBP categories:

S2.1 Aliphatic DBPs

The aliphatic DBPs are the most commonly available DBPs (with high quantity) among all the categories in the water. These DBPs can be divided into two major sub-categories as aliphatic nitrogenous DBPs (Aliphatic N-DBPs) and aliphatic carbonaceous DBPs (Aliphatic C-DBPs). The aliphatic C-DBPs are more frequent while the aliphatic N-DBPs are more toxic. The aliphatic N-DBPs are formed from water sources with a high content of dissolved organic nitrogen (DON), especially when impacted by wastewater or algae [1,2]. The acidic characteristics of water ($pH<6$) favor the formation of aliphatic N-DBPs while the alkaline characteristics ($pH>8$) favor the formation of aliphatic C-DBPs [3]. It was found that the waters that had been least affected by anthropogenic contamination contained the least quantity of DON. Furthermore, the lower the ratio of dissolved organic carbon (DOC) to DON, the greater the likelihood of the formation of Aliphatic N-DBPs [4]. Aliphatic N-DBPs are also classified as intermediate DBPs as they hydrolyze at higher pH and temperature [5]. These compounds are typically formed instantaneously during water disinfection but are prone to be dissolved when reacting with residual disinfectants or during hydrolysis reactions [6]. Haloacetamides (HAMs), Halonitromethanes (HNMs), Haloacetonitriles (HANs), and N-nitrosamines are the four major families of recognized aliphatic N-DBPs and most of them are unregulated. On the other hand, THM, HAA, and Haloacetaldehydes (HAL) are the classes of commonly investigated aliphatic C-DBPs and most of them are regulated. The broad overview of all the families of aliphatic N-DBPs and aliphatic C-DBPs is expressed in the following sub-sections.

Haloacetamides (HAMs)

The nitrogen-containing organic compounds (e.g., amino acids, pyrroles, and pyrimidines) along with the disinfectant (such as chlorine) have been associated with the formation of aliphatic N-DBPs including haloacetamides [7] (Figure S1). The majority of these compounds are identified in surface water as compared to groundwater. It is also reported that the antibiotic chloramphenicol may contribute to the formation of HAMs in heavily wastewater-impacted waters [8]. The formation of this family of DBP increases with the increase of the concentration of disinfectant (such as chlorine). In the literature, the major identified compounds for this family of DBPs are 2,2-Dibromoacetamide (DBAcAm), 2,2-Dichloroacetamide ($C_2H_3Cl_2NO$), and 2,2,2-Trichloro-acetamide ($C_2H_2Cl_3NO$) [9]. The estimated concentration of these compounds is up to $7.4\ \mu g/L$ in the treated surface water that is discharged from a plant (in the United States) [10,11]. However, many other compounds are also identified with minimal amounts. Although their concentration is lower than that of regulated DBPs (such as THMs), these compounds are more hazardous and must be controlled. These compounds are *142.2* times more toxic than aliphatic C-DBP (such as HAAs) and *1.4* times more toxic than other aliphatic N-DBPs (such as HNMs and HANs) [12]. The research for HAMs shows different cytotoxicity to two exposure pathway-related cell lines: human gastric epithelial cell line GES-1 and immortalized human keratinocyte cell line HaCaT [13].

Halonitromethanes (HNMs)
Another type of aliphatic N-DBPs is Halonitromethanes (HNMs) [14] (Figure S2). HNMs are also formed in reactions of nitrogen-containing organic compounds with a disinfectant such as chlorine [7]. It has been observed that the highest HNM concentrations were obtained with ozonation-chlorination, followed by chlorination, ozonation-chloramination, and chloramination [14]. Moreover, the hydrophilic components of NOM are most likely the primary precursors of HNMs [14]. In the literature, the major identified compounds for this family of DBPs are trichloronitromethane (TCNM) and bromonitromethane (BNM), and their estimated concentrations in the treated surface water that is discharged from a plant (in the United States and Western Australia) are up to *10 μg/L* [1,11]. These compounds are genotoxic (more) as well as cytotoxic inducing high levels of DNA signature breaks [15].

Haloacetonitriles (HANs)
HANs (as shown in Figure S3) are the most common aliphatic N-DBPs detected in the surface/groundwater. It is also one of the most frequently detected DBPs out of all the families of DBPs. Many organizations including the World Health Organization (WHO) and the United States Environmental Protection Agency US-EPA have already implemented regulations for the compounds of this family [1]. Similar to the other two families of DBPs (mentioned above), this type of DBP is formed due to the reaction of nitrogen-containing organic compounds with a disinfectant such as chlorine or ozone [7]. 2,2-dichloroacetonitrile (C_2HCl_2N) (DCAN), 2,2-dibromoacetonitrile (DBAN) (C_2HBr_2N), and 2,2,2-trichloroacetonitrile (C_2Cl_3N) (TCAN) are the most common compounds that lie in this family. The estimated concentration of HANs is considered as up to *3-14 μg/L* in the treated surface water that is discharged from a plant (in the United States) [11]. However (considering other countries such as China), DBAN with a maximum of *26.6 μg/L* was observed in post-chlorination water. Furthermore, the mean concentrations of DCAN in tap water (no treatment), boiled water (tap water after boiling), and direct drinking water (treated tap water) are *0.955 μg/L*, *0.207 μg/L*, and *0.127 μg/L*, respectively, and those of DBAN are *0.221 μg/L*, *0.104 μg/L*, *0.089 μg/L*, respectively (in China) [7,16]. DCAN is responsible for the increase of fetal resorption and reduction in fetal body weight whereas TCAN is responsible for cancer, mutagenic, and clastogenic effects [17,18]. Moreover, these compounds are also responsible for damage to the liver and kidney [16].

N-nitrosamines (NNAs)
N-nitrosamines (shown in Figure S4) can form in drinking water as a byproduct of disinfection processes that use chloramines [19]. Chloramines are commonly used as a secondary disinfectant in drinking water treatment to maintain a residual level of disinfectant in the distribution system. N-nitrosodimethylamine (NNDMA) is the major compound in this family of DBP (considering chloramines as a byproduct). NNDMA can form due to the reaction of nitrogen-containing compounds (nitrates and nitrites) with a disinfectant. This family of DBPs is extremely genotoxic, cytotoxic, and mutagenic [20]. It is important to control the formation of NNDMA in drinking

water to minimize exposure to this potential carcinogen. Government agencies already regulate the maximum limits (max.) of these compounds, such as the WHO (max. *0.1 μg/L*) [21] and Health Canada (max. *0.04 μg/L*) [22].

Trihalomethanes (THMs)

THMs are aliphatic C-DBPs that are the most frequent of all DBPs (shown in Figure S5). The compounds of this DBP family are formed when disinfectants (such as chlorine and chloramine) react with NOM such as dead plants or plant waste leaves, bush and tree clippings, animal manure, and others (DOC). They are also formed during the hydrolysis reaction of unstable DBPs such as HANs [5]. These chemicals are very volatile and permeable to the skin [23]. The most prevalent THM chemicals are trichloromethane or chloroform (CF), dibromochloromethane (DBCM), bromodichloromethane (BDCM), and tribromomethane or bromoform (BF). Organizations / unions / states already regulate the maximum limits (max.) of these compounds, such as the EU (max. *100 μg/L*) and the US (max. *80 μg/L*) [23]. Although these compounds are less harmful than N-DBPs, they are wide-spread and have severe adverse effects on the human body, prompting the necessity to impose regulations. These compounds have been found to have negative effects on the human body, including cancer (CF, BDCM, BF), liver and kidney damage (CF, BDCM, DBCM, BF), reproductive effects (CF, BDCM, DBCM), and nervous system damage (DBCM, BF) [23-25]. THMs have been detected in the blood and breath of swimmers and non-swimmers at indoor pools [26].

Haloacetic acids (HAAs)

HAAs (shown in Figure S6) are also recognized as aliphatic C-DBPs. Disinfectants such as chlorine and chloramines react with the NOM (dead plant parts, roots, living microbes, and soil animals) to generate this DBP. Furthermore, the interaction of various DBPs with chlorine yields these DBP compounds [23]. Dichloroacetic acid (DCAA), trichloroacetic acid (TCAA), monochloroacetic acid (MCAA), monobromoacetic acid (MBAA), and dibromoacetic acid (DBAA) are the five most common compounds in this family. These compounds are more commonly found in drinking water sourced from surface water compared to groundwater. Because these substances have serious human health consequences such as bladder cancer and reproductive abnormalities, they are already regulated by government authorities such as the US-EPA (upper limit *60 μg/L*). DCAA and TCAA have also been linked to cancer, liver, and kidney damage, as well as spleen and developmental consequences [24].

Haloacetaldehydes (HAL)

HAL (shown in Figure S7) is considered a member of the aliphatic C-DBP family, yet they are less common than the other two aliphatic C-DBP families. The compounds of this DBP family have been identified in groundwater (as well as in surface water) and they are generated by the reaction of disinfectants (mainly chlorine and chloramines) with NOM (mainly acetaldehyde). The number of these compounds increases as the pH and disinfectant doses increase [27]. The

compounds and their concentration (in drinking water treated with chloramines or chlorine in the United States) that are identified as HAL include tribromoacetaldehyde (TBAL) with *1-25 μg/L*, chloroacetaldehyde (CAL) with *0.25-10 μg/L*, dibromoacetaldehyde (DBAL) with *0.25-10 μg/L*, bromochloroacetaldehyde (BCAL) with *0.1-10 μg/L*, dibromochloroacetaldehyde (DBCAL) with *1-25 μg/L*, iodoacetaldehyde (IAL) with *0.5-8 μg/L*, bromoacetaldehyde (BAL) with *0.5-10 μg/L*, bromodichloroacetaldehyde (BDCAL) with *1-25 μg/L*, dichloroacetaldehyde (DCAL) with *0.25-10 μg/L*, and hydrated trichloroacetaldehyde or chloral hydrate ($TCAL\text{-}H_2O$) with *0.25-10 μg/L* [28]. Compared to other DBP families, these compounds are highly cytotoxic, with TBAL having the highest cytotoxic level and $TCAL\text{-}H_2O$ having the lowest. Furthermore, DBAL has the highest genotoxicity level while $TCAL\text{-}H_2O$ has the lowest [28].

S2.2 Alicyclic DBPs

Among the three DBP categories (aliphatic, alicyclic, aromatic), alicyclic DBPs have received the least attention. However, the investigation of the DBP families that fall into this category requires attention as these families can be considered emerging DBPs. With limited resources, the two families of alicyclic DBPs explored in the literature are halobenzoquinones (HBQ) and halofuranones. The following sub-sections provide a quick summary of these families.

Halobenzoquinones (HBQ)

HBQ (shown in Figure S8) is one of the two families of alicyclic DBPs, and it is one of the significant families that need further research as inhaling compounds from this family increases the risk of bladder cancer in humans [29,30]. The compounds in this family are generated by the reaction of a disinfectant (such as chlorine) with NOM. It has been observed that the lignin-like and highly oxygen-containing components of NOM play a significant role in the formation of HBQ [31]. These compounds are found in greater concentrations in surface water than in groundwater (ground tap water). The majority of these compounds, with concentrations (in the treated water) ranging from 0.5-275 *ng/L,* are 2,6-dichloro-3-methyl-1,4-benzoquinone (DCMBQ) and 2,3,6-trichloro-1,4-benzoquinone (TCBQ). However, the absence of any HBQ compounds was observed in the raw water. These compounds are extremely cytotoxic and may be genotoxic and carcinogenic. They can cause cellular protein and DNA damage, as well as the development of bladder cancer [29].

Halofuranones (HFur)

Halofuranones have been identified in drinking water [17] and they belong to well investigated DBP families (Figure S9). The chemical reaction of disinfectant with the NOM (such as humic substances) produces the compounds of this DBP family. 3-chloro-4-(dichloromethyl)-5-hydroxy-2-(5*H*)-furanone, also known as "mutagen X" (MX) constitutes the major compound that has been identified as halofuranone. The estimated concentration level of the compounds belonging to this family is approximately *100 ng/L* when Cl_2 is used as a disinfectant (concentration *1.9-6.9 mg/L*) in the United States [17]. The halofuranones have been shown to have genotoxicity and mutation

effects, and they are one of the most potent carcinogenic DBPs that can cause cancer in the human body [17,23]. However, the risk of DNA damaging potential of MX is lower than some other aliphatic DBPs [17].

S2.3 Aromatic DBPs

The last of the three categories of DBPs are the aromatic DBPs. This category shares a planar cyclic structure that follows Hückel's rule and occurs at a much lower concentration as compared to aliphatic DBPs [17]. The planar cyclic structure typically consists of a planar ring of sp^2 hybridized carbon atoms with alternating double bonds and single bonds. The most well-known example of an aromatic ring is benzene, which has six carbon atoms in a planar ring and satisfies Hückel's rule. NOM containing higher aromatic rings such as phenyl or heterocyclic structures may be more likely to form aromatic DBPs. The investigation of these DBPs is also important as the regulation of popular DBPs cannot fully control the adverse health effects caused by consuming disinfectant drinking water [30]. Moreover, this category of DBPs can act as a precursor of aliphatic DBPs [17].

Over *100* aromatic DBPs have been identified in swimming pools, wastewater, and drinking water [17]. The level of toxicity for the DBP family of this category is higher as compared to other regulated DBPs and it may cause an unpleasant taste or odor in drinking water [17,32]. Aromatic DBPs are divided into three classes: phenyl nitrogenous DBPs (phenyl N-DBPs), phenyl carbonaceous DBPs (phenyl C-DBPs), and heterocyclic DBPs. Phenyl N-DBPs have functional groups containing N atoms such as $-NO_2$, $-NH_2$, and $-CN$, but phenyl C-DBPs do not. Similarly, planar cyclic structures such as pyrrole and pyridine are also seen in heterocyclic DBPs [7]. The majority of the heterocyclic DBPs are unstable as they are easily hydrolyzed under alkaline conditions and can be decomposed with chlorine, whereas the phenyl N-DBPs are stable (nitrogen-containing functional groups) [7]. However, chloramine with poor oxidative capacity may result in the formation of aliphatic DBPs from aromatic DBPs. (Halo)phenylacetonitriles (HPANs) and halonitrophenols (HNP) are the two major families of recognized phenyl N-DBPs. Similarly, halophenols (HP), halohydroxybenzaldehydes (HBADs), and halohydroxybenzoic acids (HBAC) are the classes of commonly investigated phenyl C-DBPs. On the other hand, halopyrroles are considered an important family of heterocyclic DBPs. An overview of all the families of aromatic DBPs (phenyl N-DBPs, phenyl C-DBPs, and heterocyclic DBPs) is expressed in the following sub-sections.

Halophenylacetonitriles (HPANs)

HPAN (shown in Figure S10) is one of the most hazardous DBPs of all the phenyl (*N*- and *C*-) DBP families. The carboxylic group in NOM reacts with disinfectants such as chlorine to form compounds of the HPAN family. This DBP family is formed due to the decomposition of plant and animal organic matter. These contaminants have been found in drinking water, pool water, and wastewater (surface water and groundwater). The two primary compounds that belong to

HPAN and have a maximum estimated concentration of *530 ng/L* in treated drinking water in China, where Cl_2 is used as a disinfectant at a concentration of *2.5-3.0 mg/L*, are 2-chloro-phenylacetonitrile and 2,5-dichlorophenylacetonitrile [17]. These compounds are extremely cytotoxic and cause developmental effects in the human body [17].

Halonitrophenols (HNP)

HNP (shown in Figure S11) is another type of phenyl N-DBP identified in the literature, and it is the most toxic among all the families of phenyl DBPs. The compounds of this family are available in both surface water and groundwater and form mainly due to the reaction of chlorine (as a disinfectant) with the NOM. The identified four major compounds include 2,6-dichloro-4-nitrophenol, 2-bromo-6-chloro-4-nitrophenol, 2,6-dibromo-4-nitrophenol, and 2,6-diiodo-4-nitrophenol and the average concentration level is estimated as 5 *ng/L* (in drinking water treated with Cl_2 at a concentration of *0.59-0.76 mg/L* in China) [17,23]. The toxicity characteristics of this family are similar to HPANs, and it is also extremely cytotoxic and causes developmental effects in the human body [17].

Halophenols (HP)

HPs are one of the phenyl C-DBPs that are the most frequent of all phenyl C-DBPs (Figure S12). The level of toxicity of HP is higher as compared to other phenyl C-DBPs. This family's compounds are formed due to the reaction among the disinfectant (chlorine) and NOM (NOM with phenyl structures such as chlorophenol, phenols, lignin, and humic acids), and these compounds are also available in surface water and groundwater. Plenty of compounds of this family have already been identified, however, the frequently occurring compounds include 2-chlorophenol, 2,4-dichlorophenol, 2,4,6-trichlorophenol, 2-iodophenol, 4-iodophenol, 4-iodo-2-methylphenol, 2,6-dichloro-4-bromophenol, 4-hydroxy-3-iodophenol, 2,6-dibromo-4-chlorophenol, 2,4,6-tri-bromophenol, 2,4,6-triiodophenol, 4-hydroxy-3,5-diiodophenol [17]. The concentrations of these compounds range from *2.5* to *32 ng/L* in drinking water treated with Cl_2 at a concentration of *0.59 - 0.76 mg/L* in China. They are cytotoxic, causing developmental abnormalities, endocrine disturbance, and growth inhibition problems [23]. Moreover, they have been recognized as the cause and promoters of cancer and tumors in the human body [17].

Halohydroxybenzaldehydes (HBADs)

Another type of phenyl C-DBPs formed due to the reaction of disinfectants with the NOM (such as natural humic and fulvic acids) is HBADs (shown in Figure S13). The frequently identified compounds that fall into this family are 3,5-dichloro-4-hydroxy-benzaldehyde, 3-bromo-5-chloro-4-hydroxy-benzaldehyde, and 3,5-dibromo-4-hydroxy-benzaldehyde. Although these compounds are detected in surface water and groundwater, their concentration level in water is low (in *ng/L*) [17]. These compounds are cytotoxic and have developmental consequences [17,23]. Liu *et al.* also argue that the growth inhibitory effects of HBAD compounds are more than families of phenyl N-DBPs [17]. The chemical structure of HBAD is shown in Figure S13.

Halohydroxybenzoic acids (HBAC)

HBAC (see Figure S14) is the final family of phenyl C-DBPs, and their properties are similar to those of the HBAD family. 3,5-dichlorosalicylic acid, 3,5-dichloro-4-hydroxybenzoic acid, 3-bromo-5-chlorosalicylic acid, 3-iodo-4-hydroxybenzoic acid, 3,5-dibromo-4-hydroxybenzoic acid, 3,5-dibromosalicylic acid, and 4-hydroxy-3,5-diiodobenzoic acid are the most well-known compounds in this family. Compounds belonging to this family are estimated to be present at a concentration of *70.2 ng/L* in tap water treated with Cl_2 at a concentration range of *0.3-0.9 mg/L* in China [17]. Although the quantity of these contaminants in the water is relatively low (in *ng/L*), they are genotoxic and cytotoxic; they can induce growth inhibition [17,33].

Halopyrroles (HPyr)

The aromatic heterocyclic DBPs are more hazardous than phenyl and alicyclic DBPs [17]. The halopyrroles (Figure S16) are too unstable and too toxic. These compounds are also formed due to the reaction of disinfectants with the NOM (with aromatic and heterocyclic structures such as humic substances, fulvic acids, polycyclic aromatic hydrocarbons, and heterocyclic aromatic compounds). The most commonly studied compound in this family is 2,3,5-tribromopyrrole, which has an estimated concentration in treated water of 6.1×10^{-5} *mol/L* (identified in Israel as a disinfectant) [17,23]. It has been found that the compounds of this family are toxic as they have a negative effect on the developmental growth of the human body [23].

S2.4 Oxyhalide compounds

Aside from the compounds discussed above, a group of oxyhalide compounds must also be examined due to their impact on human health. They are a group of inorganic compounds that contain both oxygen and halogen atoms. The general chemical formula for an oxyhalide is XO_mZ_n, where X is a halogen atom such as chlorine, bromine, or iodine; O is an oxygen atom; Z is a metal or nonmetal atom; m and n are integers that indicate the number of atoms of each element in the compound. Examples of oxyhalides include bromate, chlorate, and chlorite [34]. Bromate can be formed by ozonating bromide-containing water or by chlorinating bromide-containing water, whereas chlorate and chlorite are found in swimming pools at very high concentrations, often in the *mg/L* range [34]. Bromate is both genotoxic and carcinogenic. Furthermore, it raises the risk of cancer. Chlorate and chlorite, on the other hand, can harm blood cells. The government agencies set a limit of maximum *10 μg/L* for bromate (by US-EPA [35]) and *700 μg/L* for chlorate and chlorite (by WHO [36]).

Supporting Information References:

(1) Liew, D.; Linge, K. L.; Joll, C. A. Formation of nitrogenous disinfection by-products in 10 chlorinated and chloraminated drinking water supply systems. *Environmental Monitoring and Assessment* **2016**, 188, 518.
(2) Lee, W.; Westerhoff, P.; Croue, J. P. Dissolved organic nitrogen as a precursor for chloroform, dichloroacetonitrile, N-nitrosodimethylamine, and trichloronitromethane. *Environmental Science & Technology* **2007**, 41, 5485-5490.
(3) Qian, Y.; Chen, Y.; Hu, Y.; Hanigan, D.; Westerhoff, P.; An, D. Formation and control of C-and N-DBPs during disinfection of filter backwash and sedimentation sludge water in drinking water treatment. *Water Research* **2021**, 194, 116964.
(4) Chu, W.; Gao, N.; Yin, D.; Krasner, S. W. Formation and speciation of nine haloacetamides, an emerging class of nitrogenous DBPs, during chlorination or chloramination. *Journal of Hazardous Materials* **2013**, 260, 806-812.
(5) Hong, H.; Xiong, Y.; Ruan, M.; Liao, F.; Lin, H.; Liang, Y. Factors affecting THMs, HAAs and HNMs formation of Jin Lan Reservoir water exposed to chlorine and monochloramine. *Science of the Total Environment* **2013**, 444, 196-204.
(6) Stefán, D.; Erdélyi, N.; Izsák, B.; Záray, G.; Vargha, M. Formation of chlorination by-products in drinking water treatment plants using breakpoint chlorination. *Microchemical Journal* **2019**, 149, 104008.
(7) Nihemaiti, M.; Le Roux, J.; Hoppe-Jones, C.; Reckhow, D. A.; Croue, J.-P. Formation of haloacetonitriles, haloacetamides, and nitrogenous heterocyclic byproducts by chloramination of phenolic compounds. *Environmental Science & Technology* **2017**, 51, 655-663.
(8) Chu, W.; Krasner, S. W.; Gao, N.; Templeton, M. R.; Yin, D. Contribution of the antibiotic chloramphenicol and its analogues as precursors of dichloroacetamide and other disinfection byproducts in drinking water. *Environmental Science & Technology* **2016**, 50, 388-396.
(9) Chu, W.-H.; Gao, N.-Y.; Deng, Y.; Krasner, S. W. Precursors of Dichloroacetamide, an Emerging Nitrogenous DBP Formed during Chlorination or Chloramination. *Environmental Science & Technology* **2010**, 44, 3908-3912.
(10) Ding, X.; Zhu, J.; Zhou, W.; Jiao, J. Disinfection by-product haloacetamides show different cytotoxicity to two exposure pathway-related cell lines: human gastric epithelial cell line GES-1 and immortalized human keratinocyte cell line HaCaT. *Environmental Epidemiology* **2019**, 3, 100.
(11) Krasner, S. W.; Weinberg, H. S.; Richardson, S. D.; Pastor, S. J.; Chinn, R.; Sclimenti, M. J.; Onstad, G. D.; Thruston, A. D. Occurrence of a new generation of disinfection byproducts. *Environmental Science & Technology* **2006**, 40, 7175-7185.
(12) Ding, S.; Chu, W.; Krasner, S. W.; Yu, Y.; Fang, C.; Xu, B.; Gao, N. The stability of chlorinated, brominated, and iodinated haloacetamides in drinking water. *Water Research* **2018**, 142, 490-500.

(13) Plewa, M. J.; Wagner, E. D.; Jazwierska, P.; Richardson, S. D.; Chen, P. H.; McKague, A. B. Halonitromethane drinking water disinfection byproducts: Chemical characterization and mammalian cell cytotoxicity and genotoxicity. *Environmental Science & Technology* **2004**, 38, 62-68.
(14) Hu, J.; Song, H.; Addison, J. W.; Karanfil, T. Halonitromethane formation potentials in drinking waters. *Water Research* **2010**, 44, 105-114.
(15) Liviac, D.; Creus, A.; Marcos, R. Genotoxicity analysis of two halonitromethanes, a novel group of disinfection by-products (DBPs), in human cells treated in vitro. *Environmental Research* **2009**, 109, 232-238.
(16) Zhang, Y.; Han, X.; Niu, Z. Health risk assessment of haloacetonitriles in drinking water based on internal dose. *Environmental Pollution* **2018**, 236, 899-906.
(17) Liu, X.; Chen, L.; Yang, M.; Tan, C.; Chu, W. The occurrence, characteristics, transformation and control of aromatic disinfection by-products: A review. *Water Research* **2020**, 184, 116076.
(18) Templeton, M.; Nieuwenhuijsen, M.; Graham, N.; Bond, T.; Huang, L.; Chen, Z. Review of the current toxicological and occurrence information available on nitrogen-containing disinfection by-products. Project No: DWI 70/2/243. *Imperial Consultants, London*, **2010**. *https://cdn.dwi.gov.uk/wp-content/uploads/2020/10/27111054/DWI70_2_243.pdf;* Accessed: 2024-02-28.
(19) Lim, S.; Lee, W.; Na, S.; Shin, J.; Lee, Y. N-nitrosodimethylamine (NDMA) formation during ozonation of *N,N*-dimethylhydrazine compounds: Reaction kinetics, mechanisms, and implications for NDMA formation control. *Water Research* **2016**, 105, 119-128.
(20) Mustapha, S.; Jimoh, T.; Ndamitso, M. M.; Abdulkareem, S. A.; Taye, S. D.; Mohammed, A. K.; Amigun, A. T. The Occurrence of *N*-nitrosodimethylamine (NDMA) in Swimming Pools: An Overview. *Environmental Health Insights* **2021**, 15, DOI: 10.1177/11786302211036520.
(21) World Health Organization (WHO). *N*-nitrosodimethylamine. History of guideline development. *https://cdn.who.int/media/docs/default-source/wash-documents/wash-chemicals/n-nitrosodimethylamine-history.pdf?sfvrsn=ae4e2701_4*, Accessed: 2024-02-28.
(22) Selin, N. Environmental Guidelines and Regulations for Nitrosamines: A Policy Summary. Massachusetts Institute of Technology, Cambridge, 10 July. 2011, *https://tcmda.com/app/uploads/sites/5/2020/09/MIT-nitrosamines-report-final.pdf*; Accessed: 2024-02-28.
(23) Kali, S.; Khan, M.; Ghaffar, M. S.; Rasheed, S.; Waseem, A.; Iqbal, M. M.; Khan Niazi, M. B.; Zafar, M. I. Occurrence, influencing factors, toxicity, regulations, and abatement approaches for disinfection by-products in chlorinated drinking water: A comprehensive review. *Environmental Pollution* **2021**, 281, 116950.
(24) Sadiq, R.; Rodriguez, M. J. Disinfection by-products (DBPs) in drinking water and predictive models for their occurrence: a review. *Science of the Total Environment* **2004**, 321, 21-46.

(25) Villanueva, C. M.; Cantor, K. P.; Grimalt, J. O.; Malats, N.; Silverman, D.; Tardon, A.; Garcia-Closas, R.; Serra, C.; Carrato, A.; Castaño-Vinyals, G.; Marcos, R.; Rothman, N.; Real, F. X.; Dosemeci, M.; Kogevinas, M. Bladder cancer and exposure to water disinfection by-products through ingestion, bathing, showering, and swimming in pools. *American Journal of Epidemiology* **2007**, 165, 148-156.
(26) Zwiener, C.; Richardson, S. D.; De Marini, D. M.; Grummt, T.; Glauner, T.; Frimmel, F. H. Drowning in disinfection byproducts? Assessing swimming pool water. *Environmental Science & Technology* **2007**, 41, 363-372.
(27) Mao, Y.; Zhang, W.; Qi, S.; Yang, H.; Xie, Y. F. Kinetics and mechanism of haloacetaldehyde formation from the reaction of acetaldehyde and chlorine. *Chemosphere* **2021**, 283, 131253.
(28) Jeong, C. H.; Postigo, C.; Richardson, S. D.; Simmons, J. E.; Kimura, S. Y.; Marinas, B. J.; Barcelo, D.; Liang, P.; Wagner, E. D.; Plewa, M. J. Occurrence and comparative toxicity of haloacetaldehyde disinfection byproducts in drinking water. *Environmental Science & Technology* **2015**, 49, 13749-13759.
(29) Li, J.; Wang, W.; Moe, B.; Wang, H.; Li, X. F. Chemical and toxicological characterization of halobenzoquinones, an emerging class of disinfection byproducts. *Chemical Research in Toxicology* **2015**, 28, 306-318.
(30) Diana, M.; Felipe-Sotelo, M.; Bond, T. Disinfection byproducts potentially responsible for the association between chlorinated drinking water and bladder cancer: A review. *Water Research* **2019**, 162, 492-504.
(31) Lou, J.; Lu, H.; Wang, W.; Zhu, L. Molecular composition of halobenzoquinone precursors in natural organic matter in source water. *Water Research* **2022**, 209, 117901.
(32) Ma, X.; Deng, J.; Feng, J.; Shanaiah, N.; Smiley, E.; Dietrich, A. M. Identification and characterization of phenylacetonitrile as a nitrogenous disinfection byproduct derived from chlorination of phenylalanine in drinking water. *Water Research* **2016**, 102, 202-210.
(33) Richardson, S. D.; Fasano, F.; Ellington, J. J.; Crumley, F. G.; Buettner, K. M.; Evans, J. J.; Blount, B. C.; Silva, L. K.; Waite, T. J.; Luther, G. W.; McKague, A. B.; Miltner, R. J.; Wagner, E. D.; Plewa, M. J. Occurrence and Mammalian Cell Toxicity of Iodinated Disinfection Byproducts in Drinking Water. *Environmental Science & Technology* **2008**, 42, 8330-8338.
(34) Guidance on the Biocidal Products Regulation European Chemical Agency (ECHA). *https://echa.europa.eu/documents/10162/15623299/bpr_guidance_vol_v_dbp_en.pdf/a57a2905-923a-5aa3-ead8-45f5c5503daf/,* Accessed: 2024-02-28.
(35) Kumar, A.; Rout, S.; Singhal, R. K. Health Risk Assessment for Bromate (BrO_3) Traces in Ozonated Indian Bottled Water. *Journal of Environmental Protection* **2011**, 2, 571.
(36) World Health Organization (WHO) Chlorite and Chlorate in Drinking water. *https://cdn.who.int/media/docs/default-source/wash-documents/wash-chemicals/chlorateandchlorite0505.pdf?sfvrsn=d844be50_5/,* Accessed: 2024-02-28.
